# Identification of the passion fruit (*Passiflora edulis* Sims) MYB family in fruit development and abiotic stress, and functional analysis of *PeMYB87* in abiotic stresses

**DOI:** 10.3389/fpls.2023.1124351

**Published:** 2023-05-05

**Authors:** Yan-shu Zhang, Yi Xu, Wen-ting Xing, Bin Wu, Dong-mei Huang, Fu-ning Ma, Ru-lin Zhan, Pei-guang Sun, Yong-yan Xu, Shun Song

**Affiliations:** ^1^ National Key Laboratory for Tropical Crop Breeding, Haikou Experimental Station, Tropical Crops Genetic Resources Institute, CATAS/ Germplasm Repository of Passiflora, Haikou, Hainan, China; ^2^ Hainan Key Laboratory for Biosafety Monitoring and Molecular Breeding in Off-Season Reproduction Regions, Sanya Research Institute, Chinese Academy of Tropical Agricultural Sciences, Sanya, Hainan, China; ^3^ Hainan Yazhou Bay Seed Laboratory, Sanya, Hainan, China; ^4^ College of Landscape and Horticulture, Southwest Forestry University, Kunming, Yunnan, China

**Keywords:** passion fruit, MYB, PeMYB87, abiotic stress, expression analysis

## Abstract

Environmental stresses are ubiquitous in agricultural cultivation, and they affect the healthy growth and development of edible tissues in passion fruit. The study of resistance mechanisms is important in understanding the adaptation and resistance of plants to environmental stresses. In this work, two differently resistant passion fruit varieties were selected, using the expression characteristics of the transcription factor MYB, to explore the resistance mechanism of the MYB gene under various environmental stresses. A total of 174 MYB family members were identified using high-quality passion fruit genomes: 98 2R-MYB, 5 3R-MYB, and 71 1R-MYB (MYB-relate). Their family information was systematically analyzed, including subcellular localization, physicochemical properties, phylogeny at the genomic level, promoter function, encoded proteins, and reciprocal regulation. In this study, bioinformatics and transcriptome sequencing were used to identify members of the PeMYB genes in passion fruit whole-genome data, and biological techniques, such as qPCR, gene clone, and transient transformation of yeast, were used to determine the function of the passion fruit MYB genes in abiotic stress tolerance. Transcriptomic data were obtained for differential expression characteristics of two resistant and susceptible varieties, three expression patterns during pulp development, and four induced expression patterns under abiotic stress conditions. We further focused on the resistance mechanism of *PeMYB87* in environmental stress, and we selected 10 representative *PeMYB* genes for quantitative expression verification. Most of the genes were differentially induced by four abiotic stresses, among which *PeMYB87* responded significantly to high-temperature-induced expression and overexpression of the *PeMYB87* gene in the yeast system. The transgenic *PeMYB87* in yeast showed different degrees of stress resistance under exposure to cold, high temperatures, drought, and salt stresses. These findings lay the foundation for further analysis of the biological functions of *PeMYB*s involved in stress resistance in passion fruit.

## Introduction

Passion fruit is a tropical–subtropical crop that consists of roughly 520 species, of which approximately 70 are edible (Cerqueira-Silva et al., 2014; Xia et al., 2021). In East Asia, the main cultivars are *Passiflora edulis* Sims and *Passiflora edulis* Degener, both of which have their own characteristics. The purple fruit has a strong flavor and a sweet and sour taste, but less resistance to adversity, while the yellow fruit is sweeter and more resistant to adversity. Passion fruit is widely cultivated around the world, but mainly in less economically and technologically developed tropical regions. Because of its short growth cycle, the area under cultivation in China and neighboring countries has grown rapidly in recent years, which has brought the total area under cultivation to nearly 44,466 ha (Xia et al., 2021), and the cultivation of passion fruit has brought significant economic benefits to the growing areas. Passion fruit is nutritious and has a high medicinal value; the whole plant has antioxidant, anti-inflammatory, antibacterial, and antifungal effects, and has positive effects on the treatment of diabetes and pulmonary fibrosis (Fonseca et al., 2022), as well as on the treatment and prevention of obesity and its complications (Lucas-González et al., 2022). Since the cultivation area of passion fruit is expanding globally, the difficulties related to its cultivation are rising as well.

Passion fruit is a typical tropical fruit. During the growth and development process, it is easy to perceive changes in response to the climate and environment. Regiana dos Santos et al. (2019) found that salt stress significantly reduced plant physiological characteristics such as the height, total chlorophyll, and stomatal conductance of *Passiflora edulis* Sims plants, which seriously affected the normal growth of the plant. Therefore, identification of stress-resistance-related functional genes and analysis of their regulatory mechanisms are of great importance for the improvement of passion fruit varieties. In this study, the PeMYB transcription factor, which has never been studied before, was used as an entry point. MYB transcription factors are one of the largest families of transcription factors in plants. The first MYB transcription factor in a plant species was discovered in maize (Amit et al., 2012), and, since then, a large number of plants have been studied for the MYB transcription factor family. The MYB transcription factors are classified into four classes because they contain different amounts of highly conserved MYB domains: 1R-MYB, 2R-MYB, 3R-MYB, and 4R-MYB. The MYB structural domain is a highly conserved peptide consisting of 50 to 53 amino acids in one repeat, with a tryptophan residue (W) between every 18 to 19 amino acid residues in the MYB structural domain sequence, whose main role is to form a hydrophobic core in the H-T-H three-dimensional structure. This core contains three α-helices connected by a turn between the second and third helix. The third helix is responsible for recognizing cis-elements in target genes, and thus regulating the expression of downstream functional genes. The conserved W residues are important for forming the hydrophobic core and maintaining the three-dimensional structure of the MYB repeat sequence (Paz-Ares et al., 1987; Ogata et al., 1995; Lipsick, 1996). This also suggests that the molecular structures and biological functions of each subgroup or branch of the MYB transcription factor family are highly conserved during evolution. For example, the MYB transcription factor *FfMYB1* in bamboo (*Fargesia fungosa*) shares high amino acid sequence similarity with Arabidopsis *AtMYB20* and *AtMYB43*, both of which are pseudo-activators of lignin synthesis (Wang et al., 2012). Buckwheat (*Fagopyrum esculentum*) *FeMYBF1* is a homology of Arabidopsis *MYB11*, *MYB12*, and *MYB111*, which all perform a regulatory role in flavonol biosynthesis (Matsui et al., 2018). Studies have shown that 2R-MYB is the most abundant subclass of the MYB transcription factor family. This subclass is widely involved in abiotic stress hormone response and secondary metabolism, such as anthocyanin flavonoid production and accumulation in plants, and cellular differentiation processes such as bud differentiation and pollen development. Some findings suggest its involvement in the biosynthesis of trophic tissue or pericarp anthocyanins, flavonoids, or lignans (Charles et al., 2018). The 3R-MYB family of transcription factors is mainly involved in the cell cycle, cell differentiation (Feng et al., 2017), and some plant stress responses, such as drought (Dai et al., 2007), low temperature (Dai et al., 2007; Zhai et al., 2010), and salt stress (Zhang et al., 2019). Mulberry (*Morus notabilis*) *MnMYB3R1* is involved in regulating the expression of its polyphenol oxidase genes (Dan et al., 2019). There are also some 3R-MYB involved in plant anthocyanin synthesis such as *MYBx1*, the R3-MYB gene of Phalaenopsis, which inhibits anthocyanin accumulation (Fu et al., 2019). In *A. thaliana*, MYB-relate was further divided into six subfamilies, consisting of 6 I-box-like, 19 TBP-like, 29 CCA1/R-type, 11 CPC-like, 2 TFR-like, and 1 orphan class (**Supplementary Table 2**), and it was found that MYB-relates act during plant growth and development to resist abiotic stresses. Thirteen percent to 65% of plant MYB-relates show substitution by alternative amino acids at the first or third W residue of the MYB structural domain (Du et al., 2013; Liu et al., 2022). In the CCA1/R-R and I-box-like subgroups, the third W residue is usually substituted by Ala (A) and Tyr (Y), respectively. In all members of the fourth branch of the Arabidopsis TBP-like subgroup, and in most members of the CPC-like subgroup, the first W residue is replaced by Phe (F). However, the shared sequences SHAQK(Y/F)F and LKDKW(R/K)(N/T) are highly conserved in the MYB domains of the CCA1-like/R-R type and TBP-like subgroups, respectively (Du et al., 2013). At present, there is little research regarding 4R-MYB, and its exact functions are still unclear. There are only two 4RMYB members in Arabidopsis and only one 4RMYB member in rice (Amit et al., 2012).

In this study, we found that the passion fruit MYB transcription factor can respond to a variety of abiotic stresses and can develop some tolerance to four abiotic stresses: salt, drought, high temperatures, and low temperatures.

## Materials and methods

### Identification of MYB genes in passion fruit, protein analysis, subcellular localization prediction

Genomic data were downloaded from the NGDC (National Genome Sciences Database), and the *PeMYB*s were initially screened and identified using hmmsearch and local blast (Output E value: 1e-5) to obtain 234 candidate MYB protein sequences. To further examine the candidate protein sequences, NCBI-CDD (https://www.ncbi.nlm.nih.gov/Structure/bwrpsb/bwrpsb.cgi), SMART (http://smart.embl-heidelberg.de/), and PFAM (https://pfam.xfam.org/) were used to remove incomplete and duplicate sequences. The 174 MYB protein sequences were then analyzed by ExPASy (http://web.expasy.org/protparam/) online software (accessed on 10 August 2021) for CDS length, protein length, molecular weight, number of isoelectric phosphate sites, and the basic physicochemical properties of the passion fruit MYB family members (Song et al., 2022).

Subcellular localization prediction was performed using http://www.csbio.sjtu.edu.cn/bioinf/Cell-PLoc-2/ (accessed on 11 August 2021).

### Construction of phylogenetic trees

A total of 126 sequences of the AtMYB protein, 5 sequences of 3R-AtMYB, and 68 sequences of the AtMYB-relate protein from *Arabidopsis thaliana* were obtained from the website (https://www.arabidopsis.org/), and 96 sequences of PtrR2R3-MYB and 5 sequences of Ptr3R-MYB from *Populus trichocarpa* were obtained from https://www.sciencedirect.com/. Multiple sequence alignments were performed using the Clustal_W tool in MEGA X (https://www.megasoftware.net/). The alignment results were used to construct a phylogenetic tree using the neighbor-joining method (NJ), Bootstrap method: 1000, Gaps/Missing Date Treatment: Partial Selection, Site Coverage Cutoff: 95. The evolutionary trees were grouped and embellished using evoview (https://www.evolgenius.info/evolview/#login).

### Gene identification and gene structure

The 174 conserved structural domains of passion fruit MYB proteins were predicted using the online software MEME (https://meme-suite.org/meme/tools/meme) with the following parameter settings: the mode was classical, the number of conserved motifs was limited to 15, and all other parameters were used as default values. Motif width was selected from 6 to 60. Finally, the results and genome structure annotation files, using MEME output clustering and analysis of conserved motifs, were obtained. The conserved motifs and exons–introns were clustered and analyzed using TBtools and plotted as a graph. The data were visualized by TBtools after performing a conserved motif search.

The conserved R-structure sequences appearing in *PeMYB*s were compared separately using MUSCLE in MEGA software, and the output was cut and manually checked using Quick Run TrimAL in TBtools. Then, each conserved fragment was visualized using the Weblogo3 online website (https://weblogo.threeplusone.com/create.cgi).

### Analysis of cis-acting elements of *PeMYB* genes

TBtools was used to obtain a 2,000-bp fragment upstream of the transcriptional start site of each *PeMYB*s and then uploaded to the PlantCARE database (accessed on 11 August 2021) (http://bioinformatics.psb.ugent.be/webtools/plantcare/html/) to identify cis-acting promoter region elements.

### Chromosomal locations

*PeMYB*s gene ID and passion fruit gff were uploaded to the TBtools “Gene Location Visualize from GTF/GFF”.

### Analysis of the protein interaction network of MYB gene in passion fruit

First, the orthovenn2 tool was used (https://orthovenn2.bioinfotoolkits.net/home) (accessed on 11 September 2021) to identify the orthologous pairs between *PeMYB*s and *AtMYB*s. Second, the interaction networks in which *PeMYB*s were involved were identified based on the orthologous genes between the passion fruit and Arabidopsis using the AraNetV2 (http://www.inetbio.org/aranet/) (accessed on 11 September 2021). The STRING (http://string-db.org/cgi) (accessed on 11 September 2021) database and the predicted interaction network were displayed using Cytoscape software (https://cytoscape.org/) (accessed on 27 September 2021).

### Gene collinearity analysis

Text Merger for MCScanX and Amazing Super Circos in TBtools were used to make an intra-species covariance analysis of the passion fruit MYB. The genome sequences of *A. thaliana*, rice, grape, and poplar were obtained with gff annotated genome files at EnsemlPlants (http://plants.ensembl.org/info/website/ftp/index.html) and NCBI Genome (https://www.ncbi.nlm.nih.gov/genome/). GXF ID Prefix was used to unify the IDs in the gff files of each species, and then One step MCscanX was used to compare the genomic files of each species. Then, Text Merge for MCScanX and Big Text View were used to merge and organize the above output files, and, finally, Multiple Synteny Plot was used to visualize the interspecies covariance of the passion fruit PeMYB genes.

The determination of selection pressure during the evolution of the MYB gene in passion fruit was performed using the TBtools Simple Ka/Ks Calculator (NG).

### Plant materials and growth conditions

Yellow-fruit passion fruit and purple-fruit passion fruit plants were selected from healthy plants after they had grown to approximately 1 m in height and had developed 8–10 functional leaves for sampling and preparation of transcriptome data for the different varieties. The growth conditions were 200 µmol·m^−2^·s^−1^; light intensity at 30°C; 12-h light/12-h dark cycle; and 70% relative humidity.

### The transcript data of *PeMYB*s analysis under different stress treatments

Healthy purple fruits were selected for the same period and sampled separately after the following stress treatments. The following stress treatments were carried out: (1) drought stress: relative soil moisture content of 50% and 10%; (2) high-temperature stress treatment: passion fruit plants were placed at 42°C for 2, 4, and 24 h; (3) low-temperature stress treatments: passion fruit plants were incubated at 4°C for 20 and 48 h; (4) salt stress treatment: passion fruit plants were watered with 300 mM NaCl liquid for 3 and 10 days. At least three leaves were collected from each treated plant, then quickly frozen in liquid nitrogen and stored in an ultra-low-temperature refrigerator. In addition, samples were taken at each of the three fruit development stages of purple-fruit passion fruit (T1, 2 weeks before harvest; T2, at harvest time; T3, 1 week after harvest) and stored in an ultra-low-temperature refrigerator (Song et al., 2022). The transcript data of the *PeMYB*s were analyzed using TBtools software (https://bio.tools/tbtools). The normalized expression data were used to generate a heatmap using TBtools.

### RNA extraction, transcriptome sequencing, and qRT-PCR

The total RNA was extracted from the frozen samples with different abiotic stress treatments and fruit maturity stages, and from the roots, stems, and leaves of the passion fruit, using a plant RNA isolation kit (Fuji, China, Chengdu) with three biological replicates. The cDNA, obtained by RT-PCR, was used for transcriptome sequencing analysis and quantitative real-time polymerase chain reaction (qRT-PCR). Primer sequences were designed using the Primer 5.0 tool. The expression of *PeMYB*s was detected by qRT-PCR analysis using SYBR^®^ Premix Ex Taq™ (TaKaRa, Japan, Tokyo). EF1α was used as the internal reference gene (Ao et al., 2022). Relative expression levels were calculated using the 2^−ΔΔCt^ method and normalized to the *PeMYB*s. The experiment was carried out using a Roche Fluorescence PCR: LightCycler 96.

### *PeMYB87* yeast (*Saccharomyces cerevisiae*) functional verification test

The full-length cDNA of *PeMYB87* was amplified from the purple-fruit passion fruit varieties by reverse transcription–polymerase chain reaction (RT-PCR) based on the sequence information in the passion fruit genome database (https://bigd.big.ac.cn/gwh/) (accessed on 1 January 2021). cDNA was extracted from healthy purple-fruit passion fruit plants. PCR products were cloned into the pMD19-T vector (Promega, Madison, WI, USA) and sequenced on an ABI PRISM310 Genetic Analyzer (PerkinElmer Applied Biosystems, Foster City, CA, USA). The full-length cDNA of *PeMYB87* was assessed by DNA MAN software. The pYES2-PeMYB87 vector was constructed using the abovementioned cloning vector and pCAMBIA1304-PeMYB87 as the amplification template by selecting two enzymatic sites, HindIII and BamHI, with specific designed primers. The constructed pYES2-PeMYB87 was transferred into the INVSC1 yeast receptor state using lithium chloride transformation in preparation for abiotic stress tolerance validation experiments. Yeast abiotic stress treatment conditions were as follows: high-temperature stress (30°C, 45°C, 50°C, 55°C, 60°C, and 65°C) was used for 2 h; salt stress (NaCl 5 mol/L); drought stress (8 mol/L PEG), treated for 2 h, 6 h, 12 h, and 24 h; and low-temperature stress (−20°C), treated for 0 h, 12 h, 24 h, 36 h, and 48 h. Tests were performed with transgenic yeast and wild-type yeast, which were induced with galactose-containing yeast induction medium.

## Results

### Physical and chemical property analysis and prediction of subcellular localization

A total of 174 *PeMYB*s sequences of the passion fruit were identified in this study. The results of physicochemical property analysis of the *PeMYB*s showed that the CDS sequence length ranged from 228 bp (*PeMYB49*) to 4,062 bp (*PeMYB172*), and the protein sequence length ranged from 75 bp to 1,353 bp (**Supplementary Table 1**). The molecular weights ranged from 8.62 kD to 149.09 kD, with an average mass of 40.05322 kD. Isoelectric point results showed PI between 4.21 (*PeMYB116*) and 11.1 (*PeMYB124*). A total of 79 PeMYB members had PI values of less than 7, and 95 had PI values higher than 7 for basic proteins. There were no neutral proteins. The predicted result of the subcellular localization showed that 11 members (*PeMYB15*/*21*/*24*/*39*/*60*/*71*/*99*/*102*/*140*/*150*/*159*) were localized in mitochondria. PeMYB110 and PeMYB138 were localized in the cytoplasm. PeMYB143 was localized in the endoplasmic reticulum. PeMYB165 was localized in the cell membrane. The other members were predicted to be localized in the nucleus (**Supplementary Table 1**). The diversity of the physicochemical property results also illustrates the structural and functional diversity of *PeMYB*s. According to the number of MYB structural domain repeats, the MYB transcription factor family was subdivided into 1R-MYB, R2R3-MYB, 3R-MYB, and 4R-MYB, and this study concluded that the largest number of *PeMYB*s was in R2R3-MYB, with 98 members, followed by 1R-MYB (71) and 3R-MYB (5). No 4R-MYB was found.

### Phylogenetic analysis of PeMYBs protein

**Figure 1:**
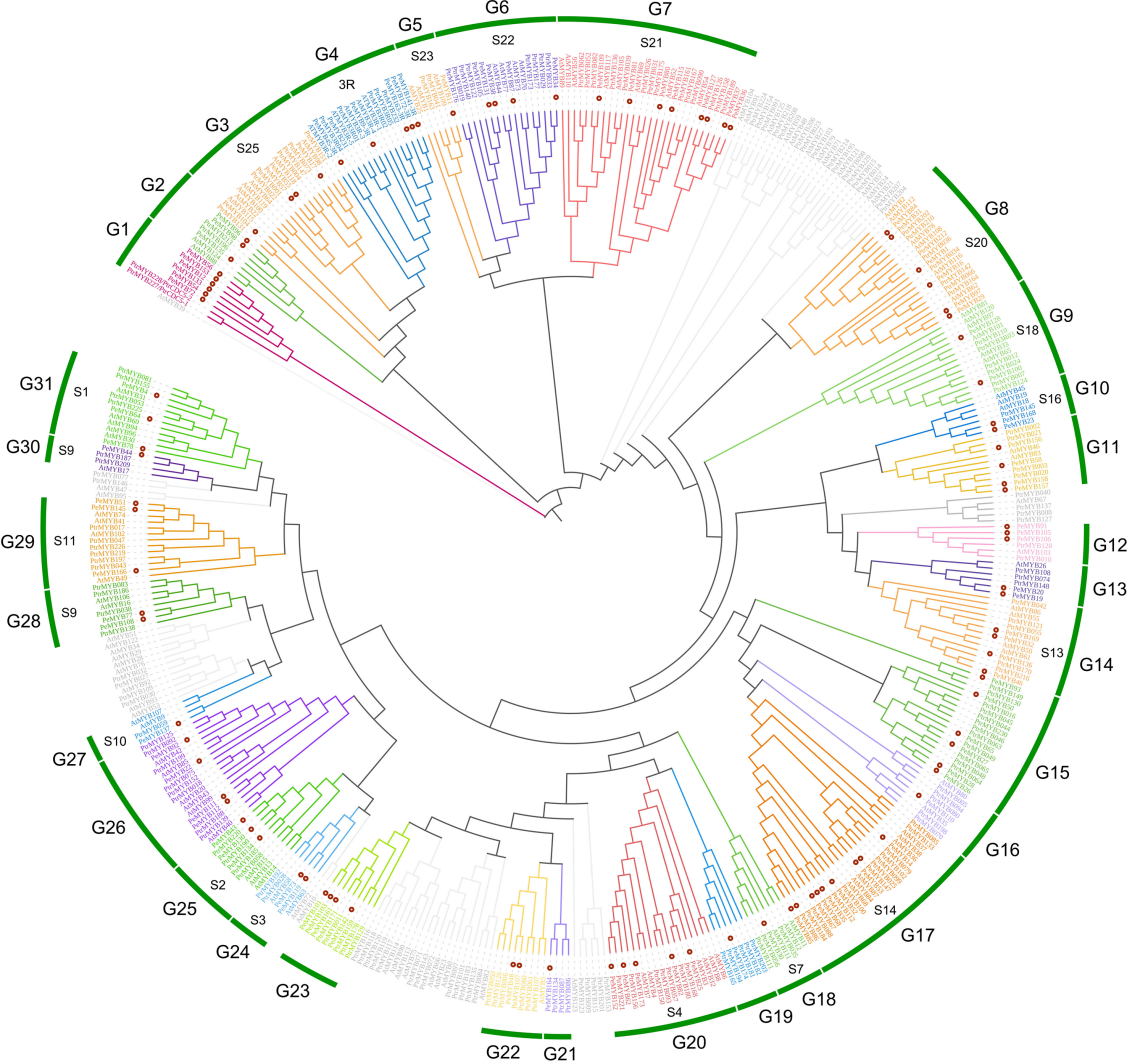
The phylogenetic evolutionary tree of PeMYB-R2R3. Ninety-eight 2R-PeMYBs proteins, five 3R-PeMYBs proteins, 126 Arabidopsis 2R-AtMYB proteins, five 3R-AtMYBs proteins, five 3R-PtrMYBs, and 196 2R-PtrMYBs proteins were compared by ClustalW. A phylogenetic evolutionary tree was generated using MEGA X and neighbor-joining methods. Red dots indicate passion fruit MYB proteins. The gray branches represent Arabidopsis or Populus trichocarpa MYB genes not clustered with passion fruit PeMYBs. The outermost circles G1–G31 represent the grouping of passion fruit, and the inner circle “S” and number combinations represent the grouping of Arabidopsis.

According to Du’s study, R2R3 class MYB may have evolved from the loss of one R3 class MYB during evolution (Du et al., 2012), and for better evolutionary analysis of *PeMYB*s, we divided the 174 members into 2R+3R and MYB-relate groups for separate phylogenetic and conserved structural domain analysis. *A. thaliana* had 126 *AtR2R3MYB* genes, 5 *AtR3MYB* genes, and 68 *AtMYB-relate* genes. A total of 196 P*. trichocarpa PtrR2R3-MYB* genes and 5 *Ptr3R-MYB* genes (Yang et al., 2021) were combined with 98 *2R-PeMYB*, 5 *3R-PeMYB*, and 71 *PeMYB-relate* (*1R-PeMYB*) group members in passion fruit to construct a phylogenetic evolutionary tree (**Supplementary Table 2**). *PeMYB*s were grouped according to 126 members of the AtR2R3-MYBs subgroup in *A. thaliana*, and, finally, the 2R-like MYBs of passion fruit were grouped into 31 subgroups, which clustered with 18 subgroups of the Arabidopsis with known functions. It was shown that MYB in the same branch may have conserved biological functions (**Figure 1**). Among these, the R2R3-like MYB of Arabidopsis has been shown to have a stress-resilient function; can be involved in the synthesis of flavonoid metabolites, epidermal waxy biology, and plant formation; and is capable of participating in the regulation of cell differentiation, cell wall, and trichome differentiation processes. The specific AtR2R3-MYB functions are shown in **Table 1**. The R3 class MYBs are grouped in a separate cluster and classified in subclade G2 (3R) (**Figure 1**). The current functions of *3R-MYB* involved in salt, drought, and cold stress also have functions in anthocyanin synthesis. The functions of these proteins in *Arabidopsis* are mainly regulation of the cell cycle and involvement in abiotic stress responses (Okumura et al., 2021). It is essential to note that the CDC5-like MYB in subgroup G1 is also found in *A. thaliana*, in addition to *P. trichocarpa*, where AT1G09770 is named AtMYBCDC5, and their amino acid sequences are extremely similar.

**Table 1:** Possible function of 2R-*PeMYB* and *3R-PeMYB*.

Group	AtR2R3-MYB	2R-PeMYB	PtrR2R3-MYB	Function	Reference
G1		PeMYB63, 72, 54, 133, 12, 56,153	PtrCDC5-1, 2		
G2	AtMYB88, 124	PeMYB96, 98, 135	PtrMYB196, 213	Regulate cell cycle, auxin regulatory genes, regulate guard cell production, transduce abiotic stress, and lateral root growth	(Yang, 2016; Xie et al., 2010; Lee et al., 2013; Chen et al., 2015)
G3 (S25)	AtMYB115, 22, 100, 64, 119, 118, AtMYB98	PeMYB104, 57, 55, 17	PtrMYB001, 022, 004, 147, 073, 151	Cellularization and differentiation during female gametogenesis; Pollen tube guidance and synergid cell differentiation, female gametophyte development	(Rabiger and Drews, 2013; Kasahara et al., 2005; Punwani et al., 2008; Susaki et al., 2021)
G4 (3R)	AtMYB3R4, 3R1, 3R2, 3R5, 3R3	PeMYB9, 45, 141, 163, 172	PtrMYB131, 3R01, 3R04, 231, 3R05, 232, 3R02	Regulate cell cycle and participate in abiotic stress	(Okumura et al., 2021)
G5 (S23)	AtMYB1, 25, 109	PeMYB11	PtrMYB163, 041	Cold resistance, regulator of tapetal and pollen development	(Verma and Burma, 2017; So et al., 2020; Beathard et al., 2021)
G6 (S22)	AtMYB77, 44, 73, 70	PeMYB38, 131, 87, 34	PtrMYB176, 019, 140, 122, 105, 033, 029, 177, 173	Stress responses and leaf senescence	(Fan et al., 2014; Nguyen and Cheong, 2018)
G7 (S21)	AtMYB89, AtMYB110, 69, 54, 117, 105, 56, 52	PeMYB109, 81, 115, 80, 126, 127, 36, 37	PtrMYB082, 031, 026, 136, 039, 062, 052, 175, 161, 167, 090, 189, 158	Lignin, xylan, and cellulose biosynthesis	(Zhong and Ye, 2009; Cao et al., 2020)
G8 (S20)	AtMYB112, 2, 108, 78, 62, 116	PeMYB31, 1, 16, 97, 29	PtrMYB210, 202, 212, 036, 164, 034, 142, 066	Anther dehiscence, ABA-responsive downstream gene, promotes anthocyanin formation, phosphate-starvation response, gibberellic acid biosynthesis	(Baek et al., 2013; Devaiah et al., 2015; Lotkowska et al., 2015; Xu et al., 2019; Sun et al., 2020)
G9 (S18)	, AtMYB120, 97, 101, 81, 65, 33	PeMYB128, 100	PtrMYB110, 012, 024, 007, 124	Anther and pollen development. Response to ABA, anoxia and cold stress, flowering, and gibberellin signaling	(Gocal et al., 2001; Leydon et al., 2013; Liang et al., 2013)
G10 (S16)	AtMYB45, 18, 19	PeMYB23, 168	PtrMYB145	Promotes photomorphogenesis	(Ballesteros et al., 2001)
G11	AtMYB46, 83	PeMYB156, 58, 158, 157	PtrMYB002, 021, 003, 020	Regulation of secondary cell wall biosynthesis	(McCarthy et al., 2009)
G12	AtMYB103	PeMYB91, 105, 106	PtrMYB128, 010	Regulates tapetum and trichome development in *Arabidopsis thaliana*. Anther growth	(Zhang et al., 2007)
G13	AtMYB26	PeMYB19, 20	PtrMYB074, 108, 148	May play a role in specifying early endothelial cell development by regulating a number of genes linked to secondary thickening	(Steiner-Lange et al., 2003)
G14 (S13)	AtMYB86, 55, 61, 50,	PeMYB169, 32, 136, 46	PtrMYB042, 121, 055, 170, 216	Anthocyanin accumulation, secondary cell wall synthesis, enhance immunity in plant, regulating stomatal size	(Liang et al., 2005; Kishi-Kaboshi et al., 2018; Sun et al., 2020; Liu et al., 2022)
G15		PeMYB93, 50, 65, 27, 28, 26	PtrMYB149, 130, 016, 045, 044, 230, 046, 063, 049, 065, 048, 064		
G16	AtMYB80, 35	PeMYB110	PtrMYB005, 094, 080, 198, 070	Cold resistance, regulator of tapetal and pollen development	(Phan et al., 2011a; Phan et al., 2011b; Feng et al., 2012; Chen et al., 2018)
G17 (S14)	AtMYB38, 37, 36, 87, 84, 68	PeMYB14, 147, 53, 52, 35, 68, 67, 86, 85	PtrMYB085, 133, 079, 102, 099, 025, 100, 088, 184	Heat resistance, drought resistance, abiotic stress, control the boundary of Arabidopsis lateral root primordium, regulate the development of trichomes and stamens	(Fernández-Marcos et al., 2016; Zhou et al., 2017; Deng et al., 2020; Zhao et al., 2020; Bewg et al., 2021; Bewg et al., 2022)
G18 (S7)	AtMYB11, 111, 12	PeMYB30	PtrMYB035, 056, 111	Flavonol, flavonol glycoside accumulation	(Stracke et al., 2007; Stracke et al., 2010; Schilbert and Glover, 2022)
G19		PeMYB107	PtrMYB203, 182, 181, 194, 165		
G20 (S4)	AtMYB8, 6, AtMYB32, 7, 3, 4,	PeMYB25, 82, 173, 62, 152	PtrMYB168, 180, 057, 093, 156, 221	Biosynthesis of phenylpropane; Increase disease resistance, flavonoid synthesis, and regulate defense traits	(Schäfer et al., 2017; Zhou et al., 2017; Kocábek et al., 2018; Ma et al., 2021)
G21		PeMYB164	PtrMYB086, 087, 134		
G22	AtMYB5	PeMYB48, 107	PtrMYB050, 126, 006, 061, 107	Differentiation of exosperm	(Gonzalez et al., 2008)
G23		PeMYB101, 103, 161, 142	PtrMYB171, 011, 159, 178, 129		
G24 (S3)	AtMYB58, 63	PeMYB73, 119	PtrMYB192, 028	Lignin biosynthesis, synthesis of secondary cell walls, cold or wound stress, plant defense	(Zhou et al., 2009)
G25 (S2)	AtMYB14, 13, 15	PeMYB43, 130, 134	PtrMYB220, 190, 185, 058	Major orchestration of shikimate, early phenylpropanoid and stilbenoid pathways, photosensitive	(Höll et al., 2013; Duan et al., 2016; Qian et al., 2020)
G26	AtMYB42, 85, 20, 43, 99, 40	PeMYB92, 22, 111, 112	PtrMYB125, 092, 199, 075, 152, 018, 188, 139	Regulates limonoids biosynthesis, regulate phenylalanine and lignin biosynthesis, abiotic stress	(Gao et al., 2013; Geng et al., 2019; Zhang et al., 2020; Yuan et al., 2021; Lin et al., 2022)
G27 (S10)	AtMYB9, 107,	PeMYB137	PtrMYB059	Suberin biosynthesis	(Lashbrooke et al., 2016)
G28 (S9)	AtMYB106, 16	PeMYB77, 108	PtrMYB083, 186, 038, 138	Cuticular wax biosynthesis, Inflorescence, pod growth, petal development, cold resistance	(Zhang et al., 2009; Jakoby et al., 2008)
G29 (S11)	AtMYB41, 102, 74, 49	PeMYB51, 145, 166	PtrMYB017, 047, 226, 219, 197, 043	Regulate the formation of stratum corneum; Salt, drought response and suberin biosynthesis	(Denekamp and Smeekens, 2003; Cominelli et al., 2008; Xu et al., 2015)
G30 (S9)	AtMYB17	PeMYB44	PtrMYB187, 209	Cuticular wax biosynthesis, Inflorescence, pod growth, petal development, cold resistance	(Zhang et al., 2009)
G31 (S1)	AtMYB31, 60, 94, 96, 30	PeMYB4, 64, 78	PtrMYB081, 155, 053, 225	Cell death during the hypersensitive response upon pathogen attack, active oxygen signal transduction, root growth, synthesis of epidermal wax	(Fichman et al., 2020; Rodríguez-Hoces de la Guardia et al., 2021; Sun et al., 2022; Lee et al., 2016)

**Figure 2:**
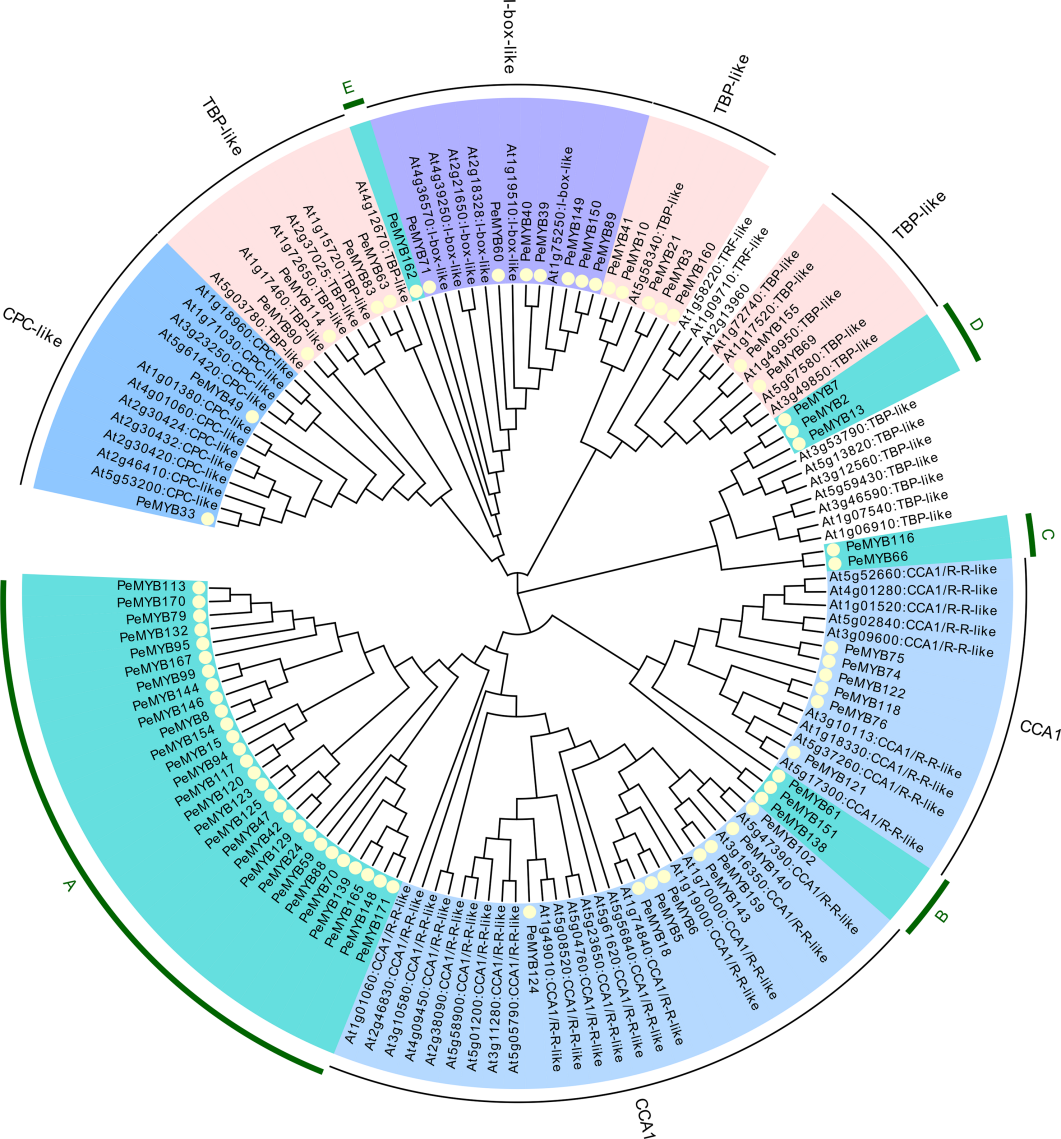
The phylogenetic evolutionary tree of PeMYB-relate. Seventy-one PeMYB-relates, five 3RPeMYB, and 68 Arabidopsis AtMYB-relates were compared by ClustalW, and a phylogenetic evolutionary tree was generated using MEGA X and neighbor-joining methods. Yellow circles indicate passion fruit MYB-relate proteins. The outermost circles A–E represent subgroups of specific passion fruit MYB-relate, and the remaining subgroups are named according to the grouping of Arabidopsis AtMYB-relate members.

MYB-related transcription factors were classified according to Arabidopsis MYB-related into 7 members of I-box-like, 2 members of CPC-like, 11 members of TBP-like, and 14 members of CCA1-like, and no TFR-like subclade was found (**Figure 2**). In addition, five independent subclusters were named A, B, C, D, and E.

PeMYB160 is a unique protein among all 71 PeMYB-relate sequences. We found that PeMYB160 appears as W(19)W(22)W in white mulberry MaMYB13 (WX19WX9PLX10W) (Liu et al., 2022). The results mainly focus on the circadian and growth hormone pathways as regulators of trichome formation, and the specific AtMYB-relate functions are shown in **Table 2**.

**Table 2:** Possible function of *PeMYB-relate*.

AtMYB-relate	PeMYB-relate	Function	Reference
CCA1/R-R-like			
At1g01060	PeMYB5, 6, 18, 74, 75, 76, 102, 118, 121, 122, 124, 140, 143, 159	Circadian clock	(Nguyen and Lee, 2016)
At1g18330		Circadian clock and auxin pathways	(Nguyen and Lee, 2016)
At1g70000		Anthocyanin biosynthesis	(Nguyen and Lee, 2016)
At2g46830		Circadian clock	(Nguyen and Lee, 2016)
At3g09600		Circadian clock by modulating the histone 3 acetylation	(Nguyen and Lee, 2016)
At5g37260		Circadian clock and seed germination	(Nguyen and Lee, 2016)
I-box-like			
At2g21650	PeMYB39, 40, 60, 71, 89, 149, 150	Required for female gametophyte development	(Pagnussat et al., 2005)
CPC-like			
At2g30420	PeMYB33, 49	Involved in epidermal cell fate specification, inhibiting non-hair cell formation	(Kirik et al., 2004)
At2g30424		Acts as a negative regulator of trichome patterning and formation.	(Gan et al., 2011)
At2g30432		Acts as a negative regulator of trichome patterning and formation	(Wang et al., 2007)
At4g01060		Negative regulator of trichome development, effects on flowering development and epidermal cell size	(Tominaga et al., 2008)
At5g53200		Promotes the formation of hair developing cells (trichoblasts) in H position in root epidermis, probably by inhibiting non-hair cell (atrichoblasts) formation	(Leydon et al., 2013)
At5g61420		Promotes aliphatic glucosinolate biosynthesis but represses indolic glucosinolate biosynthesis. Prevents insect performance	(Hirai et al., 2007)

### Gene structure of the MYB gene family of passion fruit and analysis of conserved motifs

**Figure 3:**
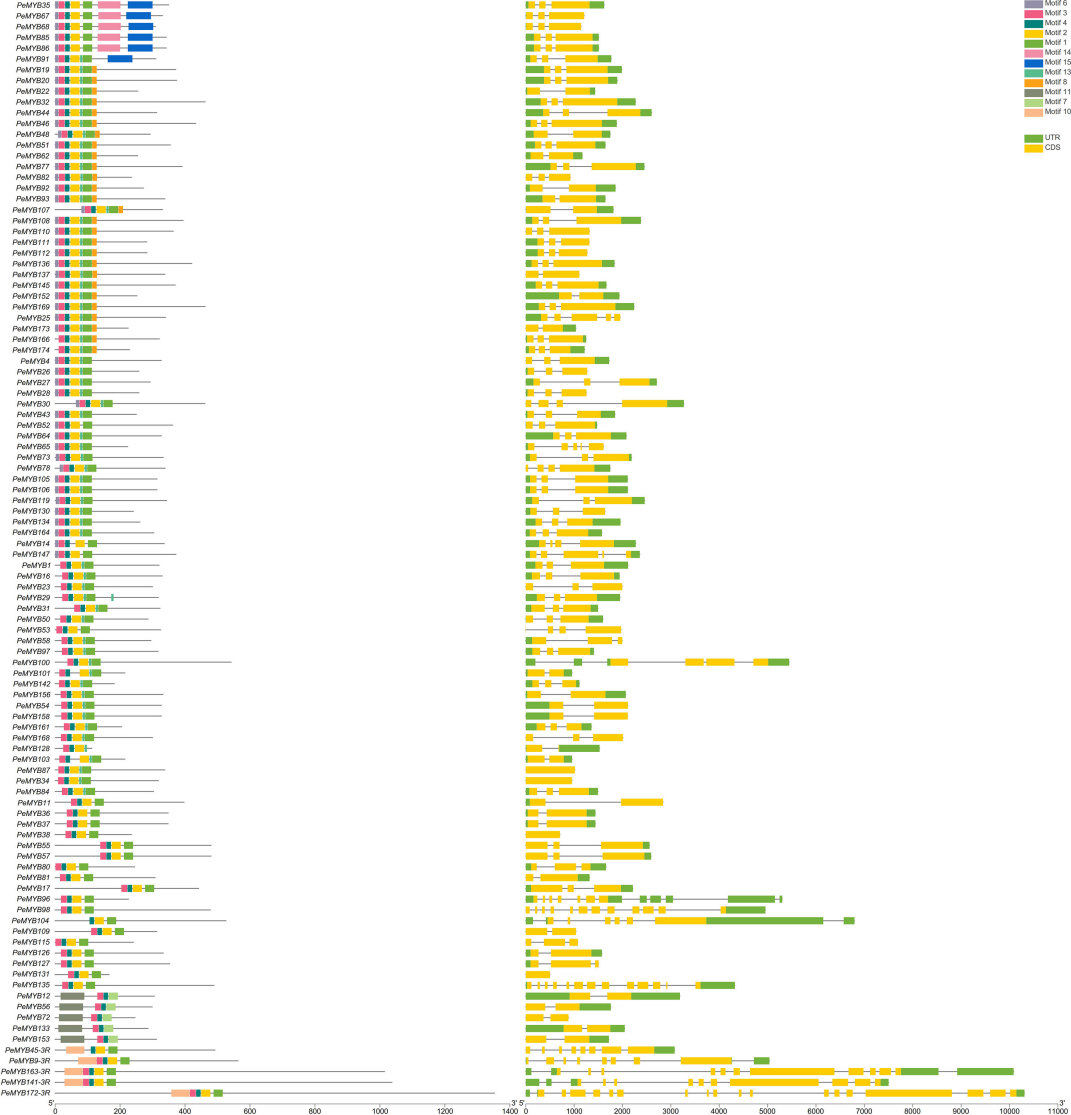
Analysis of 2R-PeMYB and 3R-PeMYB conserved structural domain and gene structure analysis. The *PeMYB*s IDs on the left side of the image are arranged according to the principle of having similar species motifs. The middle image shows the structure of the *PeMYB*s conserved protein motifs represented by blocks of different colors. The rightmost image shows the analysis of the gene structure of *PeMYB*s, with the CDS sequence in yellow and the UTR sequence in green. The length of the protein/DNA can be estimated using the scale at the bottom of the figure.

As shown in **Figure 3**, 2R-PeMYB and 3R-PeMYB members contain one to seven motifs. PeMYB35/67/68/85/86 only contain motifs 14 and 15. Motif 8 appears in 27 members, and motif 10 appears in only 5 3R-MYBs. Motifs 1, 2, and 3 appear in 98 2R-PeMYBs. Motif 1 appears in five members of PeMYB12, 56, 72, 133, and 153, and these five members also belong to PeCDC5.

The exon–intron structure is an important evolutionary feature of the gene. We further analyzed the exon–intron structures of the *PeMYB*s. *2R-PeMYB*s and *3R-PeMYB*s had, at most, seven UTRs in *PeMYB96*. *3R-PeMYB* had more CDSs with (*PeMYB45*) 8–18 (*PeMYB172*) and (*PeMYB45*) 1–4 (*PeMYB9*/*141*/*163*) UTRs. Among all *2R-PeMYB*s, *PeMYB34*/*38*/*87*/*131* contain one CDS, while the rest of the members contain two or more CDSs. A total of 13 *PeMYB*s family members contain only one 5’UTR, with no 3’UTR. The 14 members of *PeMYB4* contain only one 3’UTR and no 5’UTR.

**Figure 4:**
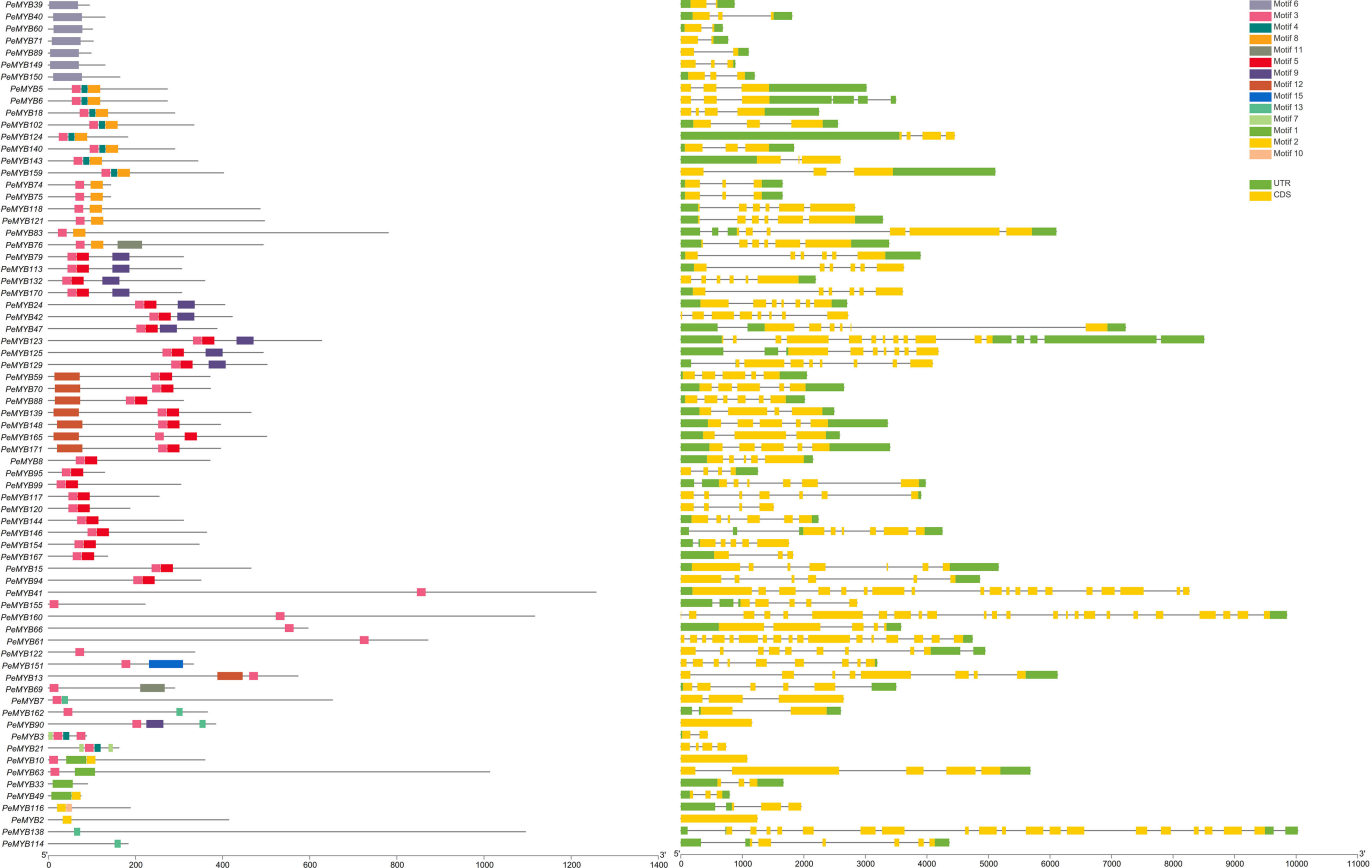
*PeMYB-relates* conserved structural domain and gene structure analysis.

**Figure 5:**
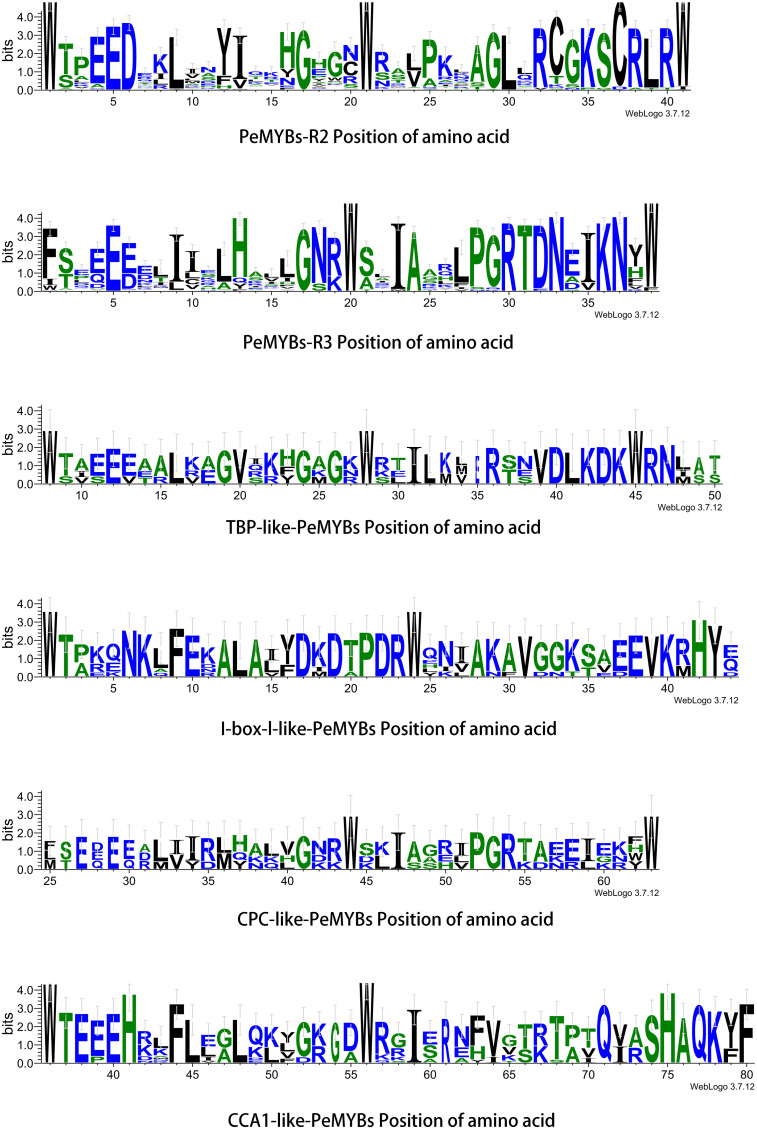
PeMYB-R structure amino acid position. Residual conservation for all proteins is shown by the height of each letter. The bit score indicates the information content of each position in the sequence. The red circles indicate five conserved tryptophan (W) residues and one phenylalanine (F/I/W) residue in the R structural domain of the MYB gene family.

The gene structures of the PeMYB-relate gene families and the results of the conserved motif analysis are shown in **Figure 4**, which indicates that motif 6 is only present in PeMYB39/40/60/71/89/149/150, which belong to the I-box-like subgroup in the phylogenetic evolutionary tree analysis, and motif 8 is present in 14 members of PeMYB5, and so on, which are part of the CCA1 subgroup. Motif 12 is present with only 7 members, including PeMYB59, and motif 9 is present with only 10 members, including PeMYB24, all of which are members of the PeMYB-related specificity subgroup. Among the *PeMYB-related* genes containing (*PeMYB2*) 1–24 (*PeMYB160*) CDS, seven members did not have UTRs, and *PeMYB123* contained the largest number of UTRs (6). **Figure 5** shows that the structure of R2 appeared relatively conserved, with three conserved tryptophan (W), while R3 repeated with F/I/M instead of the first W and Y/F instead of the third W. The third amino acid of R1 was replaced by Y/F. The analysis of the PeMYB-related-R structure is consistent with the previous results in the Arabidopsis AtMYB-related motif (Du et al., 2013).

### Analysis of the cis-acting elements of the promoter of the MYB gene of passion fruit

**Figure 6:**
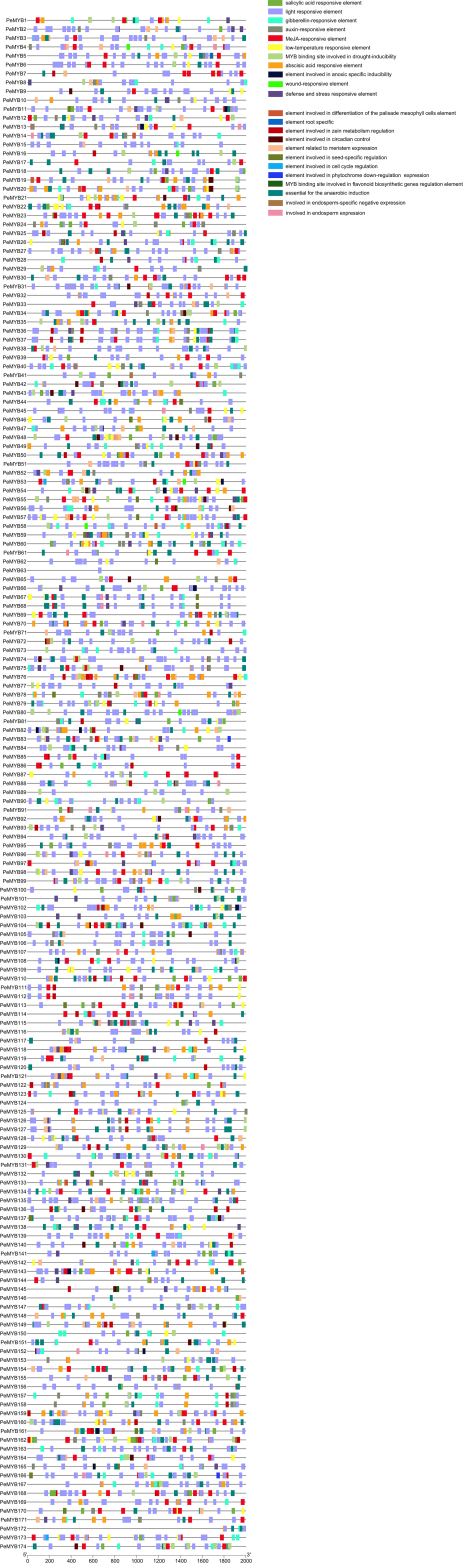
Distribution of promoter cis-acting elements for 174 *PeMYB*s. The rightmost side of the image is labeled with rectangles of different colors representing different functional cis-acting elements. Chromosomal localization analysis of the *PeMYB*s genes in passion fruit.

Analysis of the action elements of the *PeMYB*s promoter yielded a total of 4435 response elements for 12 types (**Figure 6**). The largest percentage is light response elements, with 44.51%. The next largest is the MeJA response element, with 12.86%, followed by the abscisic acid response element, with 10.42%; the gibberellin response element, with 3.59%; the drought response element, with 3.07%; the low-temperature response element, with 2.71%; the auxin-responsive element, with 2.54%; the salicylic acid response element, with 2.25%; the defense and stress response element, with 1.98%; the element involved in anoxic specific inducibility, with 0.33%; and the wound response element, with 0.15%.

In addition to the stress-related cis-regulatory elements, the *PeMYB*s contain 12 other functional elements, which are related to cell differentiation to meristematic and endosperm tissues, circadian regulation, cell cycle regulation, seed-specific regulation, zein metabolism regulation, photosensitive pigment downregulation, and flavonoid biosynthesis (**Supplementary Table 3**). This suggests that members of the *PeMYB*s may be involved in almost all relevant processes of the plant’s growth and development.

### Chromosomal distribution of *PeMYB* genes

**Figure 7:**
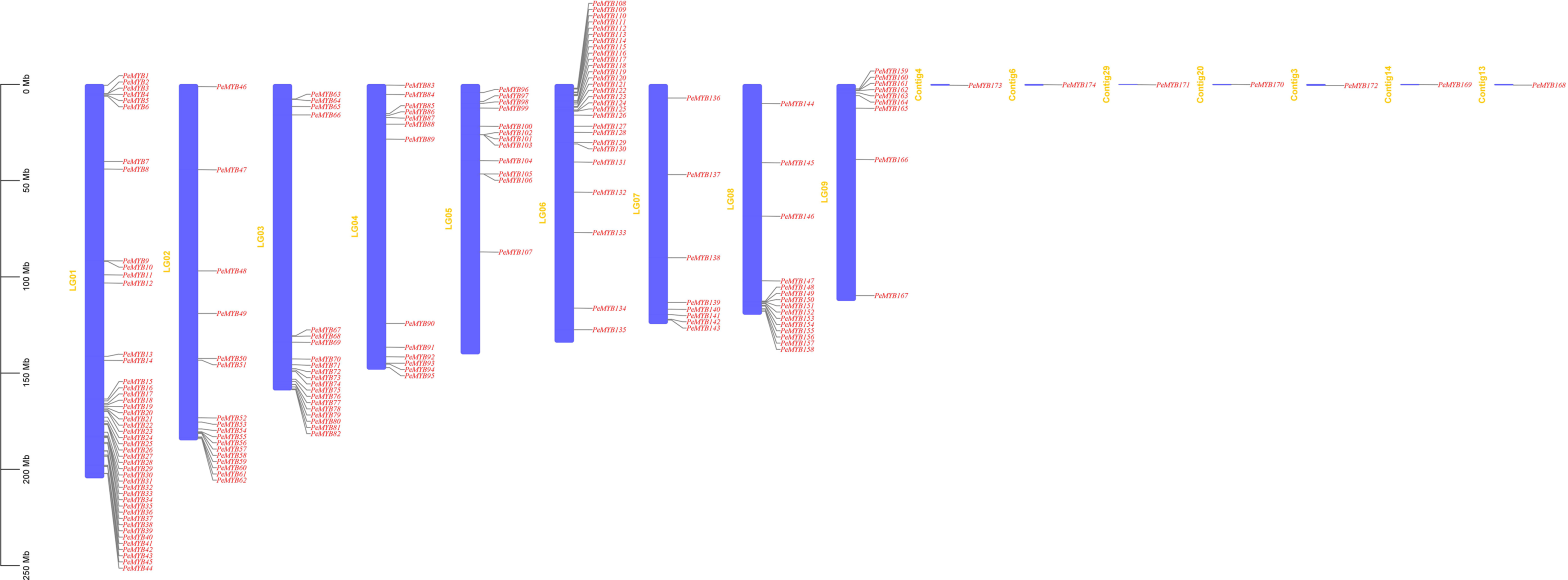
Chromosome localization analysis of PeMYB family members. The leftmost scale indicates the genetic length of the chromosomes, and the purple rectangle represents each chromosome of passion fruit. The chromosome number is located to the left of each chromosome. Scale units are megabases (Mb).

Using the passion fruit gff3 genome annotation information data, we extracted information on the chromosomal location (**Figure 7**). *PeMYB173* is on Contig 4, *PeMYB170* is on Contig 20, *PeMYB172* is on Contig 3, *PeMYB169* is on Contig 14, and *PeMYB168* is on Contig 13. There were 45 members on the Chr1(~25.86%), 17 on the Chr2 (9.77%), 20 on the Chr3 (~11.49%), 13 on the Chr4 (~7.47%), 12 on the Chr5 (~6.90%), 28 on the Chr6 (~16.09%), 8 on the Chr7 (~4.60%), and 16 on the Chr8 (~9.20%). The total number of chromosomes was 9.20%, and 10 genes were found on the ninth chromosome, accounting for 5.75% of the total.

### Analysis of the protein interaction network of MYB gene in passion fruit

**Figure 8:**
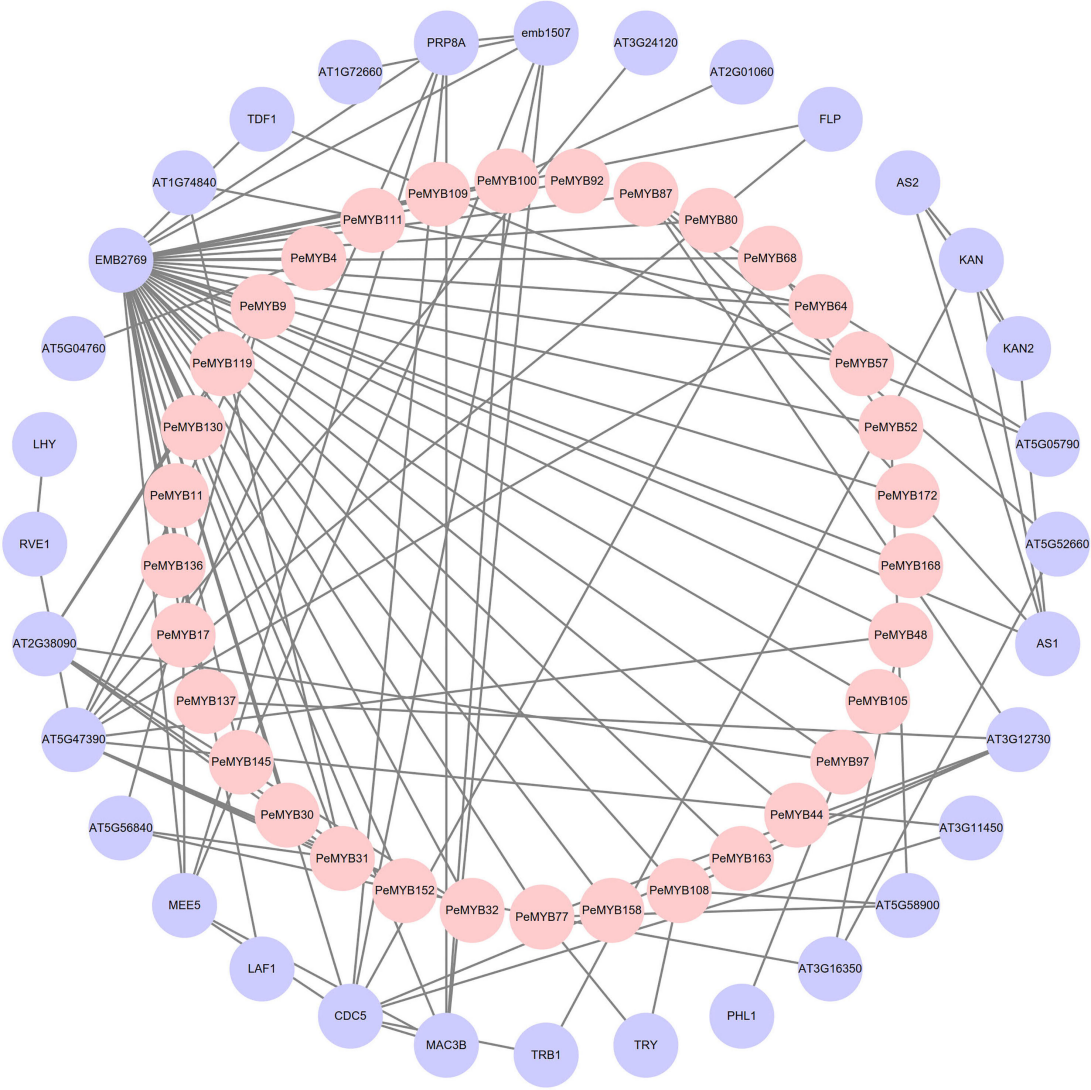
Analysis of the protein interaction network of PeMYB family members. The inner pink circles represent the passion fruit PeMYBs, and the outer purple circles represent the proteins that may interact with PeMYBs mapped in Arabidopsis.

The results of the protein interaction analysis of PeMYBs of *P. edulis* and *Arabidopsis* protein mapping are shown in **Figure 8**. There are 32 PeMYBs and 32 proteins with known functions that constitute the outer and inner circles of the protein interaction network, respectively. The outer circle of the protein AT5G47390 is involved in plant growth and development, the removal of excess reactive oxygen species from the plant, and the regulation of phytohormone synthesis (Lu et al., 2014; Huang et al., 2015). The CDC5 protein may regulate defense responses through transcriptional control and is essential for natural plant immunity. It has sequence-specific DNA sequence “CTCAGCG” binding activity. CDC5 is involved in mRNA splicing and cell cycle control. It may also play a role in the response to DNA damage (Zhang et al., 2013). FLP protein is a transcription factor that binds to DNA in the 5’-GGCGC-3’ cis-regulatory element of cell cycle gene promoters, including cell cycle proteins, cell cycle protein-dependent kinases (CDK), and components of the prereplication complex. FLP together with FAMA and MYB88 ensures that stomata contain only two guard cells (GCs) by performing a single symmetric precursor cell division prior to stomatal maturation (Yang, 2016). MAC3B may be a ubiquitin protein ligase, primarily involved in pre-mRNA splicing and DNA repair (by similarity). The components of the MAC complex that may regulate the defense response through transcriptional control are therefore essential for natural immunity in plants (Li et al., 2018). The above relationships for these proteins are consistent with existing reports on the function of MYB transcription factors in plants, suggesting that members of the MYB transcription factor family of passion fruit may also have these functions.

The above functional findings suggest that members of the *PeMYB*s may also possess these functions.

### Intra-species covariance analysis of the *PeMYB*s

**Figure 9:**
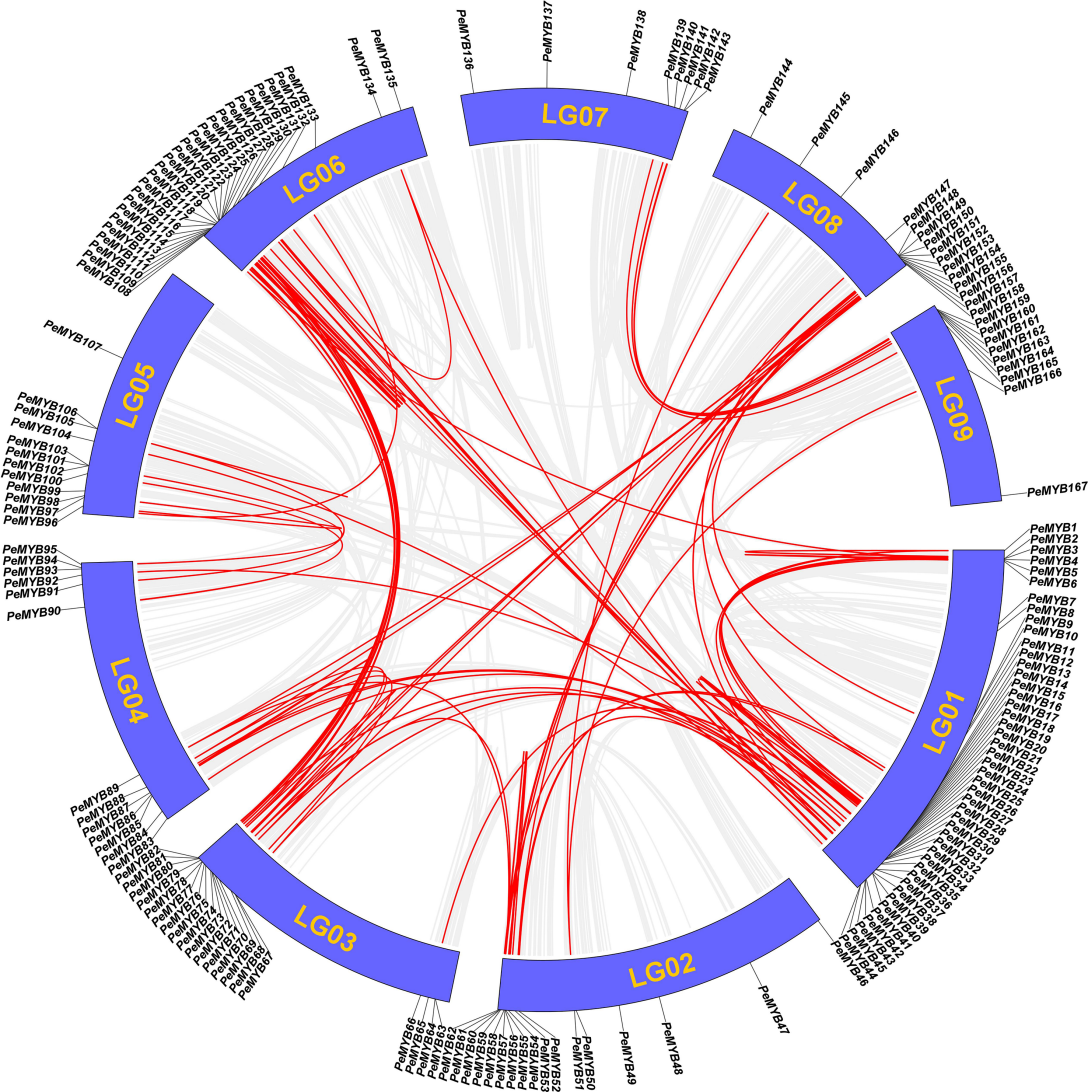
Analysis of covariance within PeMYBs species. Schematic diagram of the collinearity of PeMYBs. The gray and red lines represent all homozygous blocks and duplicated R2R2-MYB gene pairs in the passion fruit genome, respectively. Corresponding chromosome numbers are shown for each chromosome.

The result of the intra-species covariance analysis showed that there are 68 collinearity pairs involving 112 members (**Figure 9**). Approximately 64.3% of the *PeMYB*s could have been generated by duplication events, suggesting that gene duplication may have been critical to the expansion of the *PeMYB*s. Ka/Ks values were calculated by TBtools to understand the selection patterns of tandem repeat pairs in *PeMYB*s. The results showed that Ka values ranged from 0 to 0.9376, and K values ranged from 0 to 4.9523 (**Supplemental Table 4**). Notably, the 12 duplicated gene pairs had Ka/Ks values of NaN. Two gene pairs did not have Ka/Ks values because they had Ks values of 0 by manual inspection, and we found that mutations between the sequences occurred at the nucleic acid level. A total of 87 *PeMYB* gene duplication pairs had Ka/Ks ratios ranging from 0.0728 to 1.65, with only one gene pair, *PeMYB26*–*PeMYB28*, having Ka/Ks values greater than 1 (1.657), suggesting that these two genes were subjected to positive gene selection. Our examination of the amino acid sequences revealed that they differed by only one amino acid with a non-synonymous substitution. The above results suggest that the passion fruit MYB repeat genes have undergone mainly negative purifying selection during evolution, which has helped the passion fruit MYB gene family to maintain its function to some extent.

### Analysis of covariance among different species of *PeMYB*s

**Figure 10:**
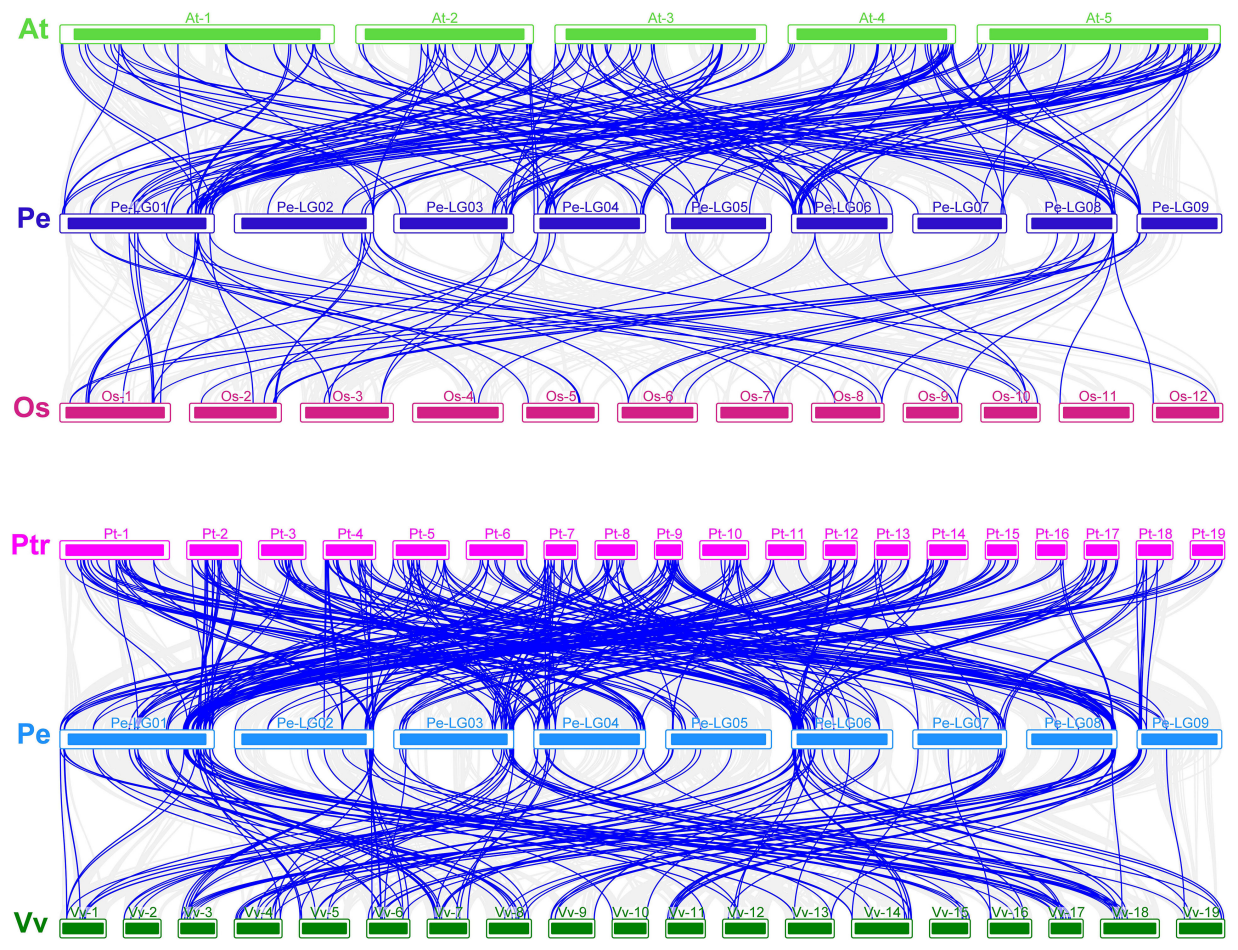
**(A)** Analysis of covariance between passion fruit and *Arabidopsis thaliana* and *Oryza sativa*. **(B)** Analysis of covariance between passion fruit, *Populus trichocarpa*, and *Vitis vinifera*. The letters on the left side of the picture are the Latin abbreviations of each species. The gray lines in the background indicate co-linear blocks in the genomes of passion fruit and other plants, while the blue lines highlight homozygous MYB gene pairs.

As shown in **Figure 10**, there were 7,473, 3,118, 25,224, and 14,638 genes associated with *A. thaliana*, *Oryza sativa*, *P. trichocarpa*, and *Vitis vinifera*, respectively, and 210, 36, 237, and 187 homologous gene pairs were identified in passion fruit, respectively. These results suggest that *P. edulis* is evolutionarily closer to *A thaliana* and *P. trichocarpa*, which are dicotyledons (Song et al., 2022).

### The transcript data of *PeMYB*s analysis under different stress treatments

**Figure 11:**
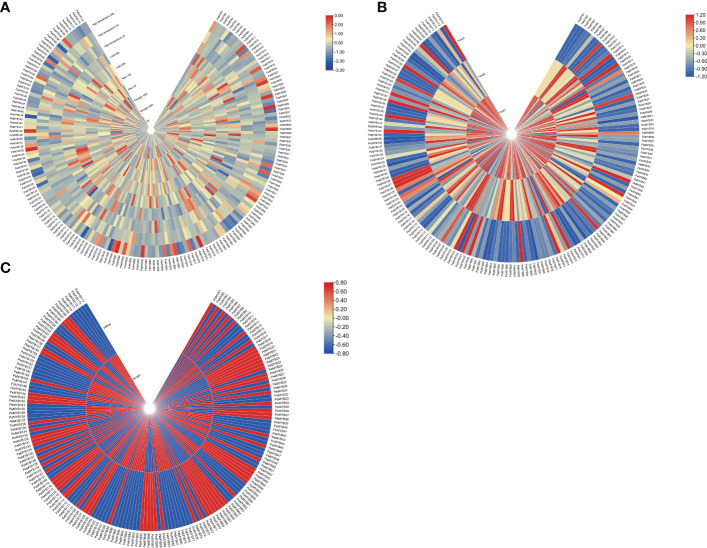
Heat map analysis of transcriptome data among PeMYB family members with different abiotic stresses **(A)**, different fruit development periods **(B)**, and different resistant varieties **(C)**. The expression profile of *PeMYB*s in passion fruit. The higher expression levels of *PeMYB*s in passion fruit are shown in blue, the higher expression levels of *PeMYB*s in the leaves of different resistant varieties are shown in blue, and the higher expression levels of *PeMYB*s in the three fruit development stages are shown in red.

The expression profiles of *PeMYBs* under various abiotic stresses were investigated using RNA-seq data (**Figure 11A**). The results showed that the *PeMYB*s have different response degrees to various abiotic stresses. Most genes were induced, and some genes were suppressed, such as *PeMYB12*/*3176*/*87*/*118*. Under salt stress, 26 genes were induced; for example, the expression levels of 10 genes, such as *PeMYB10*/*19*/*44*, were upregulated when treated with salt stress for 3 days, but reduced after 10 days. A total of 11 gene expressions were decreased by day 3 and then upregulated by day 10. Under low-temperature stress, the transcript levels of 16 genes (*PeMYB9*/*12*/*26*/*28*) were upregulated, and some were suppressed, such as *PeMYB10*/*17*/*30*/*40*. The expression levels of *PeMYB4/5*/*6*/*14*/*19*/*46*/*48*/*59*/*61*/*70*/*83*/*102/148* were suppressed when treated for 20 h and increased after 48 h. Under drought stress, *PeMYB6*/*28*/*43*/*125*/*128*/*129*/*133*/*137*/*141*/*145*/*150*/*154*/*159*/*165* expressions upregulated as the stress level intensified. *PeMYB122*/*129*/*140*/*153*/*170* expressions increased and then downregulated with the increase in drought stress. Under high-temperature stress, the transcript levels of *PeMYB8*/*12*/*34*/*38*/*41*/*42*/*43* were upregulated. These results suggest that most of the *PeMYB*s are involved in and closely related to the stress resistance process. This may also indicate that passion fruit MYB genes are widely involved in abiotic stresses and could play an influential role in future passion fruit genetic breeding studies.

We also performed the transcript sequencing of three different fruit ripening stages (T1, T2, and T3) (Xia et al., 2021), from which we analyzed the expression levels of all *PeMYB*s. The results showed that all genes were expressed in three periods, and the expression of most genes was higher at T1 and T2. However, there were a few genes with higher expression at T3, such as *PeMYB9* and *PeMYB13* (**Figure 11B**).

A total of 77 MYB genes were found to be more highly expressed in yellow fruit than in purple fruit (**Figure 11C**). A total of 17 members, such as *PeMYB 2*/*45*/*52*, were only expressed in purple fruits. However, 11 members (*PeMYB16*/*26*/*43*) were only expressed in yellow fruit. These results suggest that there are still significant differences in the expression of MYB transcription factors between the two cultivars.

### Expression patterns of *PeMYB*s under different abiotic stresses

**Figure 12:**
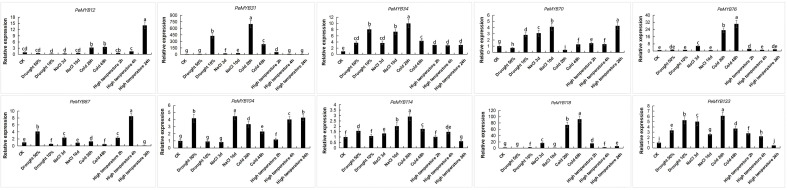
Expression analysis of 10 *PeMYB*s under abiotic stress. The error bars indicate the standard error of three replicates. Statistical significance of the difference in expression between control and treated groups was analyzed using GraphPad software. Data are means ± SD of *n* = 3 biological replicates determined by Duncan’s multiple range test, with different letters indicating significant differences in expression means at *p* < 0.05.

Ten *PeMYB*s with differentially expressed genes under different stress treatments were selected and verified by qRT-PCR. The results showed that the trend of expression is consistent with the transcriptome sequencing analysis (**Figure 12**; **Supplementary Table 5**), and the expressions of *PeMYB31*/*34*/*70*/*87*/*104/114/133* were changed under drought stress. *PeMYB34/76/87/104/114/118/133* responded to salt treatment. The genes that responded to cold treatment were *PeMYB12*/*31/34/70/76/87/104/114/118/133*, and the genes that responded to high temperatures were *PeMYB12/31/34/70/87/104/114/118/133*. The qPCR results were consistent with the trend of the transcriptome data, which also suggested that *PeMYB*s might function in the stress resistance of passion fruit.

### *PeMYB87* yeast (*S. cerevisiae*) functional verification test

**Figure 13:**
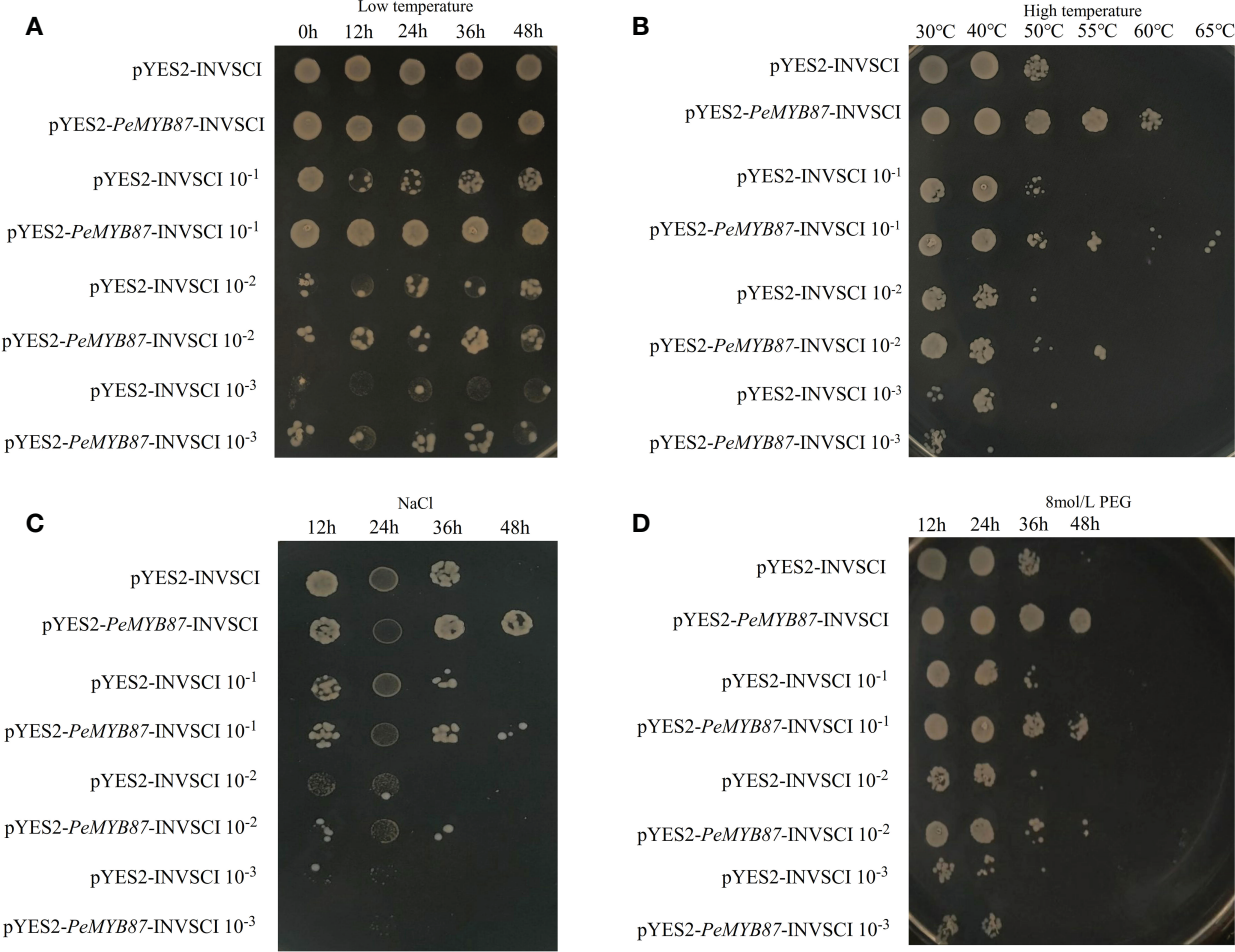
The temperature and time of treatment are marked at the top of the picture, and the left side marks represent the different dilutions of yeast solution at 1, 10^−1^, 10^−2^, and 10^−3^, respectively. **(A)** Low temperature stress. **(B)** High temperature stress. **(C)** Salt stress. **(D)** Drought stress.

The stress-tolerant gene can be expressed in the model organism, yeast (Jia et al., 2021). In this study, the *PeMYB87* gene was selected for the functional validation test of yeast. The results (**Figure 13**) showed that yeast transfected with the *PeMYB87* gene developed varying degrees of stress resistance, including high temperature, drought, and salt stresses. In addition to low-temperature stress, transgenic yeast of *PeMYB87* grew better than the control under three other abiotic stresses, and transgenic yeast colonies performed best in withstanding high-temperature stress.

## Discussion

MYB transcription factors have a diverse functional identity and can participate in regulatory networks that span almost the entire plant growth cycle (Allan and Espley, 2018); therefore, they are also the focus of functional studies of plant genes. Numerous studies of MYB transcription factors have already been published. A total of 155 and 198 MYB transcription factors have been found in two model plants, Arabidopsis and rice, respectively. A total of 256 MYB proteins were identified in peach (*Prunus persica*), namely, 128 R2R3MYB, 4 3R-MYB, 109 MYB-realted, 1 4R-MYB, and 14 atypical MYBs (Zhang et al., 2018). The MYBs of watermelon [*Citrullus lanatus* (Thunb.) Matsum and Nakai] were shown to contain 1 Cla3R-MYB, 89 ClaR2R3-MYB, and 72 ClaMYB-related genes (Xu et al., 2018). In tomatoes, 122 SlR2R3-MYB, 4 Sl3R-MYB, and 1 Sl4R-MYB were identified (Li et al., 2016). A total of 174 MYB transcription factors were identified in this study, of which 98 were 2R-MYB, 5 were R3-MYB, and 71 were MYB-relate; no 4R-MYB were identified. These results are similar to the results in *P. trichocarpa*, where 196 R2R3-MYB, 152 MYB-relate, 5 3R-MYB (mostly R2R3), and no 4RMYB were identified. In our screening analysis for members of the passion fruit MYB family, we identified an amino acid sequence with four repetitive MYB conserved structural domains (**Supplementary Figure 1**), but manually deleted it because of its missing start codon. In the future, additional sequences for the screening of 4R-PeMYB may be performed as genome sequencing and splicing technology advances. As seen in the phylogenetic tree analysis, above (**Figures 1**, **2**), they are also extremely similar to Arabidopsis in terms of clustering, which may be due to their close kinship (Yang et al., 2021). At the same time, passion fruit MYB also produced specific clustering, which may be caused by the loss of the last ancestor of passion fruit and Arabidopsis after separation during the long evolutionary process (Xu et al., 2018), resulting in *PeMYB*s containing the conserved MYB structural domain as well as other, more conserved, motifs. The motif structures shown in **Figure 4** also explain their failure to aggregate with Arabidopsis AtMYB-related members. In our study of PeMYB-relate phylogenetic analysis, the grouping we used was based on the six subgroups assigned to *A. thaliana*, and, in other research, there is a finer classification based on the different functions of the MYB-related genes. In the next in-depth study of the functions of *PeMYB-relate*d genes, we will make a finer classification. A number of studies have demonstrated that plant MYB genes have functions to resist stresses. For example, R2R3-MYB transcription factor *RmMYB108* responds to low-temperature stress in *Rosa multiflora* and confers cold resistance in Arabidopsis (Dong et al., 2021); overexpression of *Fragaria vesca* MYB transcription factor gene (*FvMYB82*) improves salt tolerance and cold tolerance in Arabidopsis (Li et al., 2022); and R2R3-MYB transcription factor *ZmMYB31* in maize positively regulates CBF gene expression and enhances resistance to cold and oxidative stress (Li et al., 2019), among others.

In this study, the results of the violin plot of the transcriptomic data generated under passion fruit stress are shown in **Supplementary Figure 2**, where the upper quartile and lower quartile values of MYB gene expression increased to different degrees with the intensification of various stresses, and the expression of most MYB changed. The upper edge of the violin plot under each stress was higher than the upper edge of the CK value, which may be the result of different levels of stress stimulating the expression of certain genes to increase them, and thus involve them, in the process of resisting the adverse environment. These genes will be the main targets of our future studies on the function of *PeMYB* transcription factors in resisting adversity.

A statistical analysis of transcriptome data from three passion fruit ripening periods using the Biozeron cloud platform (http://www.Cloud.biomiclass.com/CloudPlatform) yielded eight significantly different expression trends (**Supplementary Figure 3**), with the number of genes generating these eight trends accounting for 3.45%, 4.59%, 10.34%, 10.92%, 14.37%, 18.39%, 27.58%, and 10.34% of the total number of genes, respectively. Among them, trends one, six, seven, and eight are the highest expressed in the first period; two, three, and four are the highest in the middle period; and five is the highest in the third period. Passion fruit undergoes significant changes in the amount of its internal metabolites during ripening (Xia et al., 2021), and MYB transcription factors are very closely related to the metabolism and synthesis of plant flavonoids, which increase the accumulation of flavonoid substances by regulating the expression of key genes related to the flavonoid biosynthesis pathway, which, in turn, can inhibit the accumulation of reactive oxygen species (ROS) (Baba and Ashraf, 2019; Wang et al., 2019). As mentioned in the Introduction section, yellow-fruit passion fruits have higher sweetness and are more resistant to abiotic stresses than purple-fruit passion fruits. MYB transcription factors are involved in fructose accumulation and catabolism in addition to resisting stress, and can regulate fructose-active enzymes (FAZYs)-mediated regulation of sucrose: sucrose 1-fructosyltransferase (1-SST), fructan: fructan 1-fructosyltransferase (1-FFT), and fructan 1-exohydrolases 1, 2a, and 2b (1-FEH1, -2a, and -2b) (Wei et al., 2017a) in a regulatory network of source libraries involved. The peach PpTST1 gene can encode a vesicular membrane sugar transporter protein that regulates sugar accumulation in peach fruit, and its promoter has a cis-element that binds to MYB (Peng et al., 2020). The changes in the distribution of oligofructans after drought stress were correlated. In the heat map analysis of the transcriptome data from different passion fruit species (Wei et al., 2017b), we identified several *PeMYB* genes that were only expressed in yellow fruits, and whether these genes could play a role in the glycan metabolism of yellow fruits is a major area of future research. Among the *PeMYB*s promoter transient action elements studied above, a large number of light-responsive elements appeared in close association with the phenylpropanoid metabolic pathway (Hartmann et al., 2005). The high frequency of photoreactive elements in the analysis of *PeMYB*s cis-reactive elements suggests that passion fruit MYB transcription factors are likely involved in pigment synthesis, such as anthocyanin synthesis. *PeMYB32*/*169*/*136*/*46* are clustered with *AtMYB86*/*55*/*61*/*50*, whose function is accretion. These functions are important in plant resistance to abiotic stresses. For example, the R2R3-MYB-like transcription factor PFG3 enhances the resistance of plants to drought stress by promoting the accumulation of flavonoids (Baozhu et al., 2022). In the construction of the phylogenetic tree, *PeMYB30* was associated with *AtMYB11*, *AtMYB111*, and *AtMYB12*, three Arabidopsis functions related to the accumulation of flavonol and flavon glycosides. The results of the protein interaction network analysis provide an important reference for exploring the functional validation of key proteins during plant life events. In the next study, we will integrate the transcriptome sequencing results and the results of the biotransformation analysis to select key genes and use the available biological techniques to deepen the analysis of abiotic stress resistance in passion fruit. The results are based on the mapping of known functions of Arabidopsis genes to *PeMYB*s, which will allow us to build on the results of this analysis to more specifically explore more the *PeMYB* genes, and thereby to elucidate the important steps affecting the growth and development of passion fruit. In the future, we will further validate the function of the *PeMYB*s gene using yeast monohybrid, yeast dihybrid, and luciferase assays. Environmental requirements for growth, such as temperature, soil moisture, and light, are also very stringent. Therefore, it is necessary to select and breed resistant varieties of passion fruit and conduct targeted breeding studies in order to cope with possible future climatic fluctuations.

## Conclusion

In this study, we identified a total of 174 *PeMYB*s genes based on the whole-genome data of passion fruit and performed an *in silico* analysis of the PeMYB family, including physicochemical properties, subcellular localization, gene and cis-acting element structure, and chromosome localization, using bioinformatics tools (TBtools). A phylogenetic analysis and a protein interaction network analysis were also performed using model plants and closely related species to predict the function of *PeMYBs*. The biological function of the *PeMYBs* genes involved in stress resistance was resolved based on expression patterns from transcriptome data of resistant varieties, abiotic stresses, and fruit development stages. RT-qPCR was also used to verify the induced expression of 10 *PeMYB* genes under four abiotic stresses, and their differential expressions were consistent with the transcriptome sequencing results. In addition, *PeMYB87* was transformed into yeast and analyzed for its biological function. The results showed that the transformed yeast produced significant resistance to high temperatures, drought, and salt stress. These findings set the stage for future in-depth studies of the function of *PeMYB*s in passion fruit and genetic breeding efforts.

## Data availability statement

The passion fruit Genomic Data and Raw RNA-Sequence Data have been deposited in (https://ngdc.cncb.ac.cn/search/?dbId=gwh&q=GWHAZTM00000000), (https://ngdc.cncb.ac.cn/omix/release/OMIX563), accession number is OMIX563-20-01 and NCBI SRA number: SRP410034, and accession number: SRR2240515-20. The qPCR Raw Date of ten *PeMYBs* are in **Supplementary Table 5**. The *PeMYBs* transcriptome sequencing data appearing in the text are shown in **Supplementary Table 6**.

## Author contributions

Experiments were performed by Y-SZ, YX, W-TX and F-NM and SS. D-MH, BW, P-GS, R-LZ and Y-YX analyzed the data. Y-SZ and YX drafted the manuscript. YX and SS supervised the experiments and finalized the manuscript. All authors contributed to the article and approved the submitted version.
