# Supplementary figures and images for: Identification of the passion fruit (Passiflora edulis Sims) MYB family in fruit development and abiotic stress, and functional analysis of PeMYB87 in abiotic stresses
Source: Front Plant Sci. 2023 May 5;14:1124351. doi: 10.3389/fpls.2023.1124351 (PMC10196401; doi:10.3389/fpls.2023.1124351)

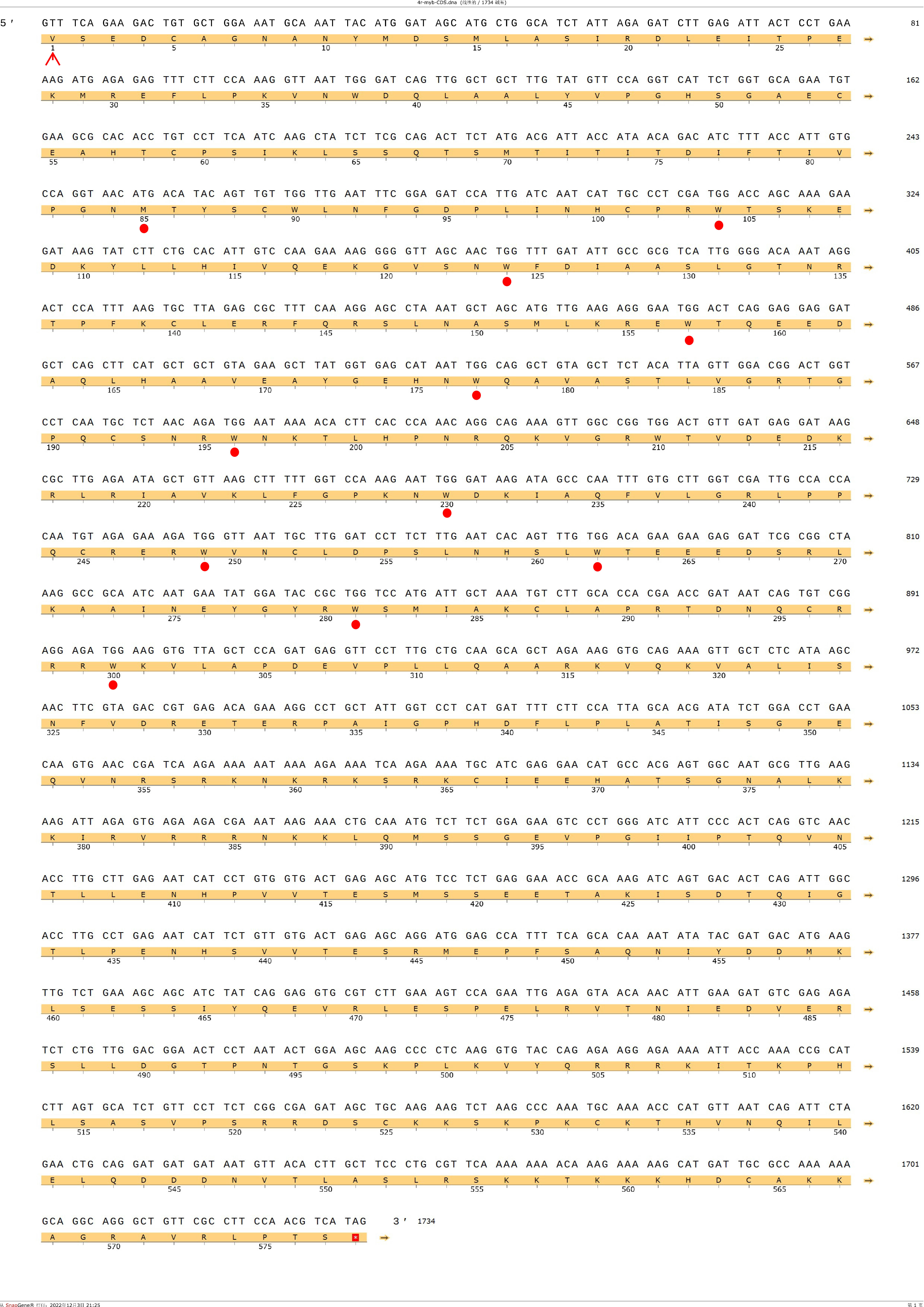

Supplement: Supplementary file 1 [file Image_1.jpg]

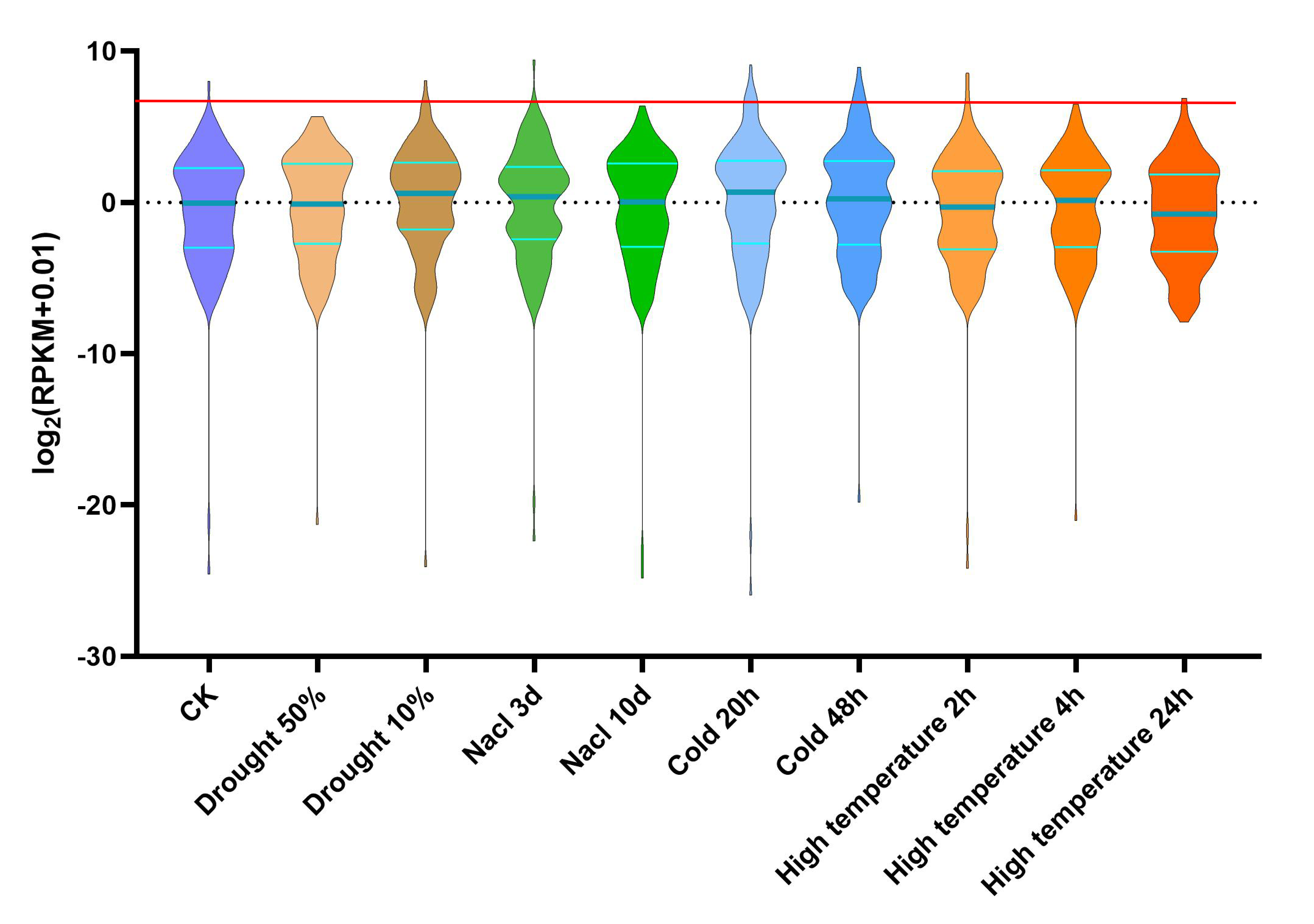

Supplement: Supplementary file 2 [file Image_2.tif]

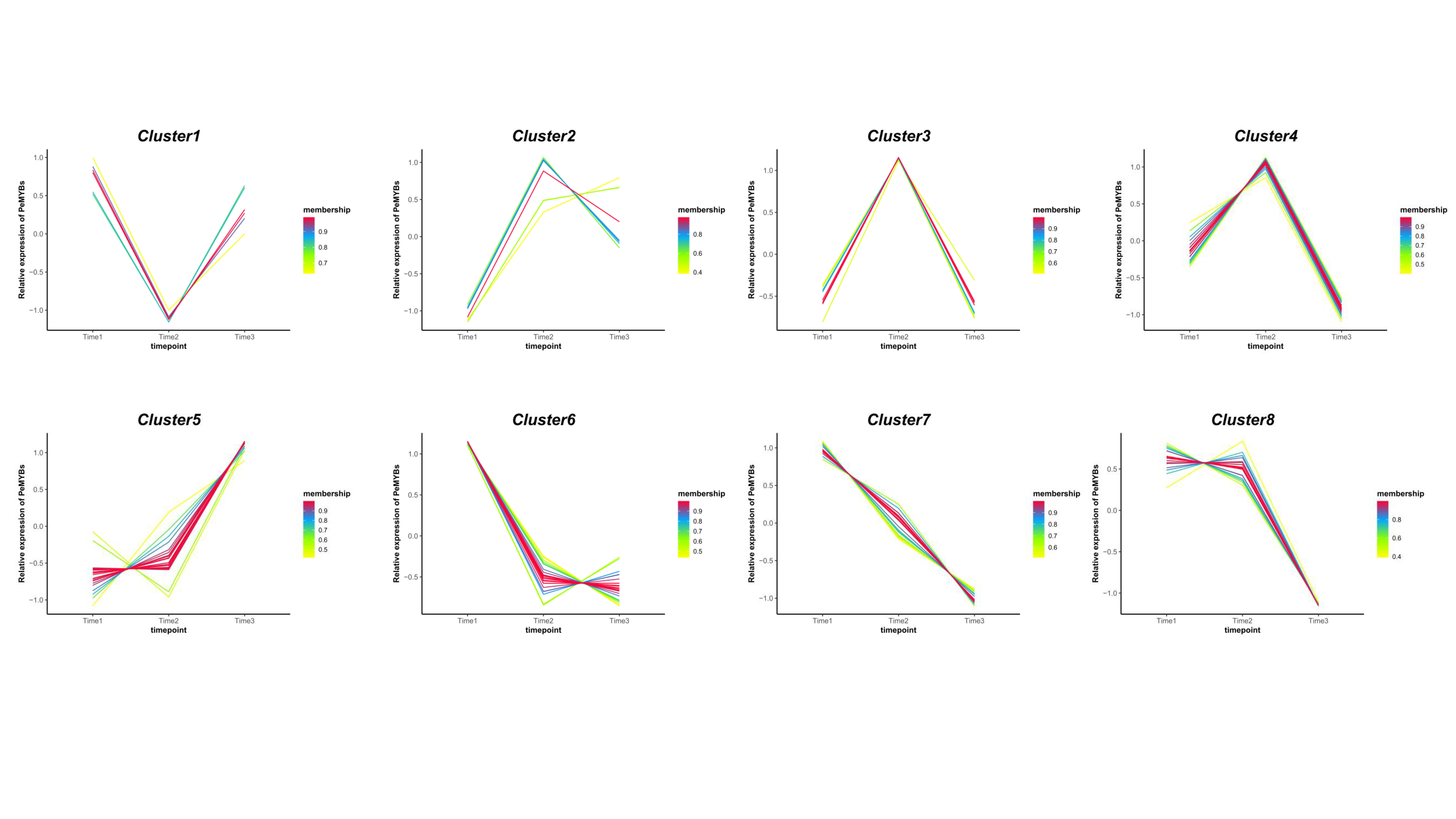

Supplement: Supplementary file 3 [file Image_3.jpg]
